# Protease mimicry: Dissecting the ester bond crosslinking mechanics in bacterial adhesin proteins

**DOI:** 10.1002/pro.70238

**Published:** 2025-07-26

**Authors:** Yuliana Yosaatmadja, Vanessa Ung, Xinlu Liu, Yixuan Zhao, Julia K. Wardega, Aria Shetty, Sophie Schoensee, Ivanhoe K. H. Leung, Jeremy R. Keown, David C. Goldstone, Edward N. Baker, Paul G. Young, Davide Mercadante, Christopher J. Squire

**Affiliations:** ^1^ School of Biological Sciences The University of Auckland Auckland New Zealand; ^2^ Maurice Wilkins Centre for Molecular Biodiscovery The University of Auckland Auckland New Zealand; ^3^ School of Chemical Sciences The University of Auckland Auckland New Zealand; ^4^ School of Chemistry and Bio21 Molecular Science & Biotechnology Institute The University of Melbourne Parkville Victoria Australia; ^5^ School of Life Sciences University of Warwick Coventry UK

**Keywords:** bacterial adhesin domain, convergent evolution, enzyme mechanism, ester bond, immunoglobin‐like domain, intramolecular crosslinking, serine protease

## Abstract

The ester bond crosslink discovered within bacterial adhesin proteins offers a captivating insight into the convergent evolution of enzyme‐like machinery. Crystal structures reveal a putative catalytic triad comprising an acid–base–nucleophile combination and an oxyanion‐like site that suggests a serine protease‐like mechanism drives the crosslinking process. We now provide confirmation of the mechanism, revealing functional catalytic dyads or triads, and the recapitulation of protease machinery from a *Pseudomonas* bacterium and a human cytomegalovirus related only by convergent evolution. Molecular dynamics simulations suggest how a conservative threonine‐to‐serine mutation of the nucleophile induces hydrolysis and eliminates the ester bond crosslink. Collectively, our structural, functional, and computational efforts detail the molecular intricacies of intramolecular ester bond formation and underscore the convergent evolutionary adaptations of bacteria in exploiting enzyme‐like machinery to protect essential adhesin proteins from the mechanical, biological, and chemical hostilities of their replicative niche.

## 1 INTRODUCTION

One of the first and the most critical steps in pathogenic bacterial infection is the adhesion of the pathogen to the host cell using long, filamentous surface appendages (Krachler and Orth 2013; Pizarro‐Cerda and Cossart 2006; Stones and Krachler 2016). Both Gram‐negative and Gram‐positive bacteria secrete surface adhesins that comprise single multidomain proteins or monomeric components for surface assembly of pili or fimbriae (Kline et al. 2010; Proft and Baker 2009; Sauer et al. 2000). Bacterial adhesins are key players in mediating host interaction and surface adhesion (Baker et al. 2015).

In Gram‐positive bacteria, “sticky” adhesin domains are located at the tip of hair‐like pili or fimbriae that decorate the bacterial surface (Kang and Baker 2012; Proft and Baker 2009; Ton‐That and Schneewind 2004). Unlike the pili of Gram‐negative bacteria that are multimeric protein assemblies held together by extensive non‐covalent interactions (Walczak et al. 2014), Gram‐positive bacterial pili often consist of small Ig‐like domains that are arranged like “beads on a string” held together by intramolecular covalent bonds (Ton‐That and Schneewind 2004). These Gram‐positive bacterial pili are narrow in diameter (20–30 Å) and are essentially one protein molecule wide when compared to their multimeric Gram‐negative counterparts that measure 60–80 Å in diameter (Craig et al. 2006; Kang and Baker 2012; Proft and Baker 2009; Waksman and Hultgren 2009; Walczak et al. 2014). While these Gram‐positive molecules are extremely thin, they can withstand persistent proteolytic, mechanical, and thermal stress during adhesion and host colonization (Dufrene and Viljoen 2020; Stones and Krachler 2016). Central to this ability are non‐canonical covalent intramolecular crosslinks between protein side chains forming isopeptide, thioester, and ester bonds that impart remarkable stability enhancement to single adhesin protein domains or are involved directly in covalent adhesion to host cells (Alegre‐Cebollada et al. 2010; Kang et al. 2007; Kang and Baker 2009; Kwon et al. 2014; Lei et al. 2021; Miller et al. 2018; Pointon et al. 2010; Walden et al. 2015).

While intramolecular isopeptide crosslinking in such domains requires only three amino acids buried in a hydrophobic environment, the ester bond equivalent has what appears to be a full complement of the catalytic machinery of a serine protease enzyme, with up to six residues promoting nucleophilic attack between normally unreactive amino acid side chains (Blow et al. 1969; Carter and Wells 1988; Dodson and Wlodawer 1998; Hedstrom 2002; Kang et al. 2007; Kwon et al. 2014; Polgar and Bender 1969; Schwarz‐Linek and Banfield 2014). This configuration of enzyme‐like features is conserved in the several X‐ray crystal structures of homologous adhesin domains from Gram‐positive bacteria (Table S1, Supporting Information), including human pathogens [*Clostridium perfringens*, PDB ID 4NI6 and 4MKM (Kwon et al. 2014); *Mobiluncus mulieris*, PDB ID 5U5O and 5U6F (Young et al. 2023)], human oral commensal bacteria (*Gemella* sp., PDB ID 7UC3 and 8F9L) (Young and Squire 2020), and animal intestinal microbiota (*Enterococcus columbae* [pigeon], PDB ID 7UI8 (Young and Squire 2020); *Suipraoptans intestinalis* [pig], PDB ID 8F90 and 8FHA).

**FIGURE 1:**
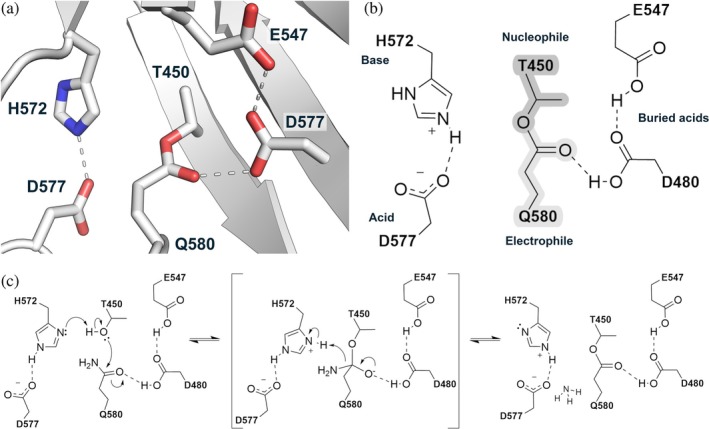
The autocatalytic site of Cpe0147. (a) The arrangement of amino acid side chains around the intramolecular ester bond crosslink site in *C. perfringens* adhesin domain (PDB ID 4NI6). The ester crosslink is formed in an autocatalytic reaction between a threonine and a glutamine that is proposed to follow a serine protease‐like mechanism. (b) 2D representation of the autocatalytic site with side chains labeled for their proposed functional role in catalysis as acid, base, nucleophile, or electrophile. The buried acids are equivalent to the oxyanion site of a serine protease and are proposed to stabilize a tetrahedral intermediate as well as the final crosslinked structure as shown. (c) The original ester bond crosslinking mechanism proposed by Kwon et al. (Kwon et al. 2014). Dashed lines represent hydrogen bond interactions that may facilitate proton transfer during catalysis.

The autocalytic machinery of ester bond crosslinking, as first exemplified in the *Clostridium perfringens* adhesin Cpe0147, shows three residues (D577/H572/T450) arranged in space to mimic the acid/base/nucleophile catalytic triad of a serine protease, with Q580 the equivalent of a substrate electrophile (Figure 1a,b) (Kwon et al. 2014; Schwarz‐Linek and Banfield 2014). Two buried and protonated acids, D480 and E547, act like the oxyanion site of a serine protease, polarizing the carbonyl bond of Q580 side chain to increase its electrophilic potential and reactivity, and potentially stabilizing a high‐energy tetrahedral intermediate (Figure 1b). In the proposed mechanism (Figure 1c), H572 abstracts a proton from T450 Oγ1, which then effects nucleophilic attack on the Q580 Cδ with the loss of the side chain amino group as ammonia—the final state is the equivalent of the acyl‐intermediate of a serine protease mechanism, but without the potential to hydrolyze it is essentially trapped. The replacement of the nucleophile threonine with a serine produces a crosslink species that is then hydrolysable at basic pH, and we have further proposed that this reaction follows the second half of a serine protease mechanism, whereby a water molecule attacks and breaks the ester bond of the acyl‐intermediate (Young et al. 2017).

While this proposed ester bond‐forming mechanism is compelling, is it a genuine example of convergent evolution of enzymatic function—the independent emergence of similar active site architectures, such as catalytic triads, and comparable mechanistic strategies in unrelated (non‐homologous) proteins? Using biochemical, biophysical, crystallographic, and computational studies, we can now reconcile previous experiments and validate a mechanism of ester bond formation in *Clostridium perfringens* Cpe0147 and homologous adhesin domains from Gram‐positive bacteria. In the process of delineating the functional role of each amino acid of the proposed catalytic machinery, we have recapitulated the functional catalytic machinery of two atypical serine proteases, one from *Achromobacter parvilus* T1, a *Pseudomonas* bacterium, and the other from *Human β‐herpesvirus 5*. The collective evidence strongly supports our proposition that the ester bond crosslinking machinery featured in bacterial adhesin domains is a bona fide example of convergent evolution of enzymatic mechanism tuned to bacterial virulence and survival.

## 2 RESULTS

Our original site‐directed mutagenesis studies described in Kwon et al. used a single Ig‐like domain comprising the first repeat domain of the 12‐domain *C. perfringens* adhesin protein Cpe0147 (Kwon et al. 2014). That study produced mutants without an ester bond crosslink that appeared unfolded by both differential scanning fluorimetry and circular dichroism. In the current study and following a closer examination of crystal structures PDB ID 4NI6 (Cpe0147^298‐438^) and PDB ID 4MKM (Cpe0147^292‐587^), we designed a new construct comprising the second Ig‐like domain (Cpe0147^439‐587^). This construct was used as the basis for a series of site‐directed mutagenesis experiments guided by established catalytic mechanisms in both conventional and atypical serine proteases. Specific amino acid functionalities were chosen for their potential to act as nucleophiles, acids/bases, or hydrogen bond donors/acceptors, while alanine substitutions were introduced to eliminate such functionality. The constructs were subjected to electrophoresis, X‐ray crystallography experiments, and finally to molecular dynamics simulations to support the hypothesis of a serine protease‐like mechanism of ester bond formation.

### 2.1 Changes to catalytic residues replicate the catalytic machinery of bacterial and viral serine proteases in nature

**FIGURE 2:**
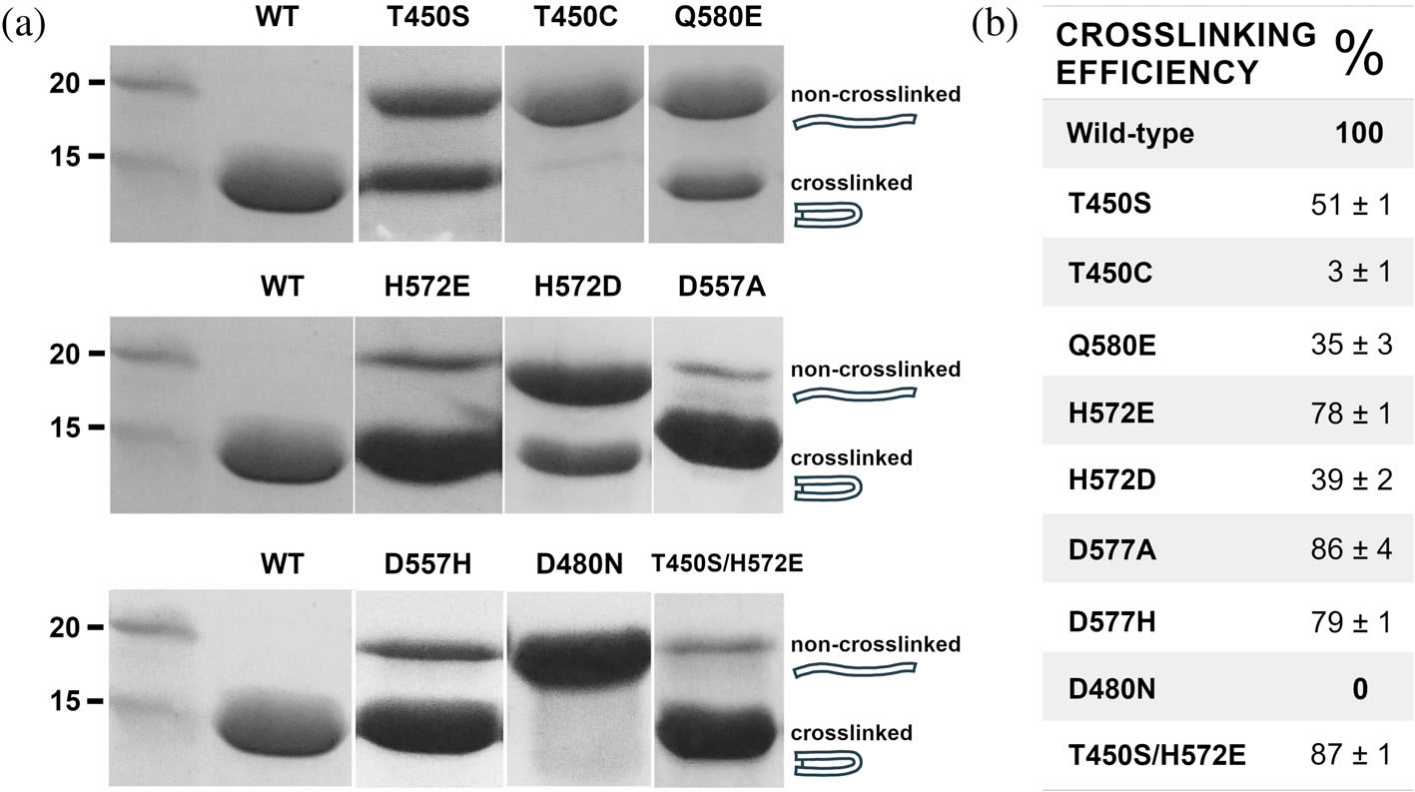
The effect of mutation on ester bond formation in Cpe0147. (a) SDS‐PAGE analysis of Cpe0147^439‐587^ protein mutants relative to wild type (WT). Non‐crosslinked protein migrates to ~20 kDa and crosslinked protein to below 15 kDa apparent mass as indicated (migration order was previously confirmed by mass spectrometry) (Kwon et al. 2014). Non‐crosslinked and ester/thioester bond crosslinked species are indicated by a linear or cyclic icon to highlight their relative size/compactness and hydrodynamic radius, which influence their migration distance. (b) Crosslinking efficiency—percentage of ester/thioester bond crosslink formed—as estimated by densitometry analysis of SDS‐PAGE data using ImageJ.

Mutations were made in wild‐type Cpe0147^439‐587^ protein to the proposed catalytic triad residues D557/H572/T450, the “substrate” Q580, and buried acids D480/E547, that correspond respectively to acid/base/nucleophile, electrophile, and oxyanion site features of a serine protease. Crosslinking efficiency was estimated from densitometry analysis of SDS‐PAGE experiments, with the mutations producing viable catalytic machinery with activity between 0% and 87% relative to wild type (Figure 2).

In chymotrypsin and trypsin‐like serine proteases, the aspartic acid within the catalytic triad is proposed to have multiple roles (Blow et al. 1969; Craik et al. 1987; Kasserra and Laidler 1969). We proposed a similar set of roles for D577 in Cpe0147. To investigate this further, we produced alanine and histidine mutations of this aspartic acid (D577A and D577H). Both mutants produce an ester bond crosslink efficiently at between 79% and 86% of wild type (Figure 2). The D557A mutant suggests that a catalytic dyad is sufficient for catalysis. This is reiterated in the D557H protein that pairs two histidines as acid/base and replicates the His/His/Ser catalytic machinery of *Human β‐herpesvirus 5* (human cytomegalovirus, HCMV) protease where a dyad was also demonstrated to be sufficient for catalysis (Figure S1b) (Chen et al. 1996; Qiu et al. 1996; Shieh et al. 1996; Tong et al. 1996).

Mutation of the second putative triad residue H572 to alanine was previously shown to abolish ester bond formation and we suggested that this histidine was acting as both base and acid in catalysis (Kwon et al. 2014). We now show that a glutamate can take the place of this histidine and retain up to 87% of the wild‐type crosslink efficiency (Figure 2; H572E, T450S/H572E). The T450S/H572E mutant reinforces the catalytic potential of a triad by replicating nature in the Asp/Glu/Ser catalytic triad of the serine protease sedolisin, found in *Pseudomonas* bacteria (Figure S1a) (Wlodawer et al. 2001). We searched the UniProt database (Consortium TU 2024) for other occurrences of a glutamate as putative base in predicted ester bond adhesin domains. The search terms “VafE repeat‐containing” and “T‐Q ester bond” identified more than 1700 entries containing multiple ester bond repeat domains. Two entries for bacterial adhesins from *Agathobacter rectalis* and an unclassified *Lachnospiraceae* bacterium present an asparagine as the “acid” equivalent of D577 paired with a glutamate as base (Figure S2).

The nucleophile identity is sensitive to change, with 100% crosslink formation in the wild‐type threonine system dropping to 51% with a conservative serine substitution and further lowering to 3% for a cysteine substitution (Figure 2). T450S Cpe0147 has been characterized in detail previously as having a reversible crosslink that hydrolyzes under specific buffer conditions. The mixture of species observed in Figure 2 may represent a dynamic equilibrium between crosslinking and hydrolysis (Young et al. 2017). The T450C protein is found predominantly as non‐crosslinked and disulfide crosslinked species under non‐reducing SDS‐PAGE (Figure 2)—this scenario is detailed below in the SEC‐MALLS and X‐ray crystallography results. The electrophile glutamine, when mutated to glutamate (Q580E) and paired with wild‐type nucleophile threonine, produces 35% crosslinked species (Figure 2).

Our final mutagenesis experiments probed the putative oxyanion site formed by the two buried acids, D480 and E547, that are proposed to stabilize a tetrahedral oxyanion intermediate via a series of hydrogen bonds or a proton shuttle arrangement. In previous mutagenesis studies, a D480A protein does not form the ester bond (Kwon et al. 2014). Our D480N mutant also displayed a complete inability to form the ester crosslink (Figure 2). Taken together, these results suggest the absolute requirement of a buried protonated acid or oxyanion‐like feature to effect autocatalysis in our crosslinking domains.

### 2.2 The dynamic behavior of T450C in solution is linked to inefficient thioester crosslink formation

**FIGURE 3:**
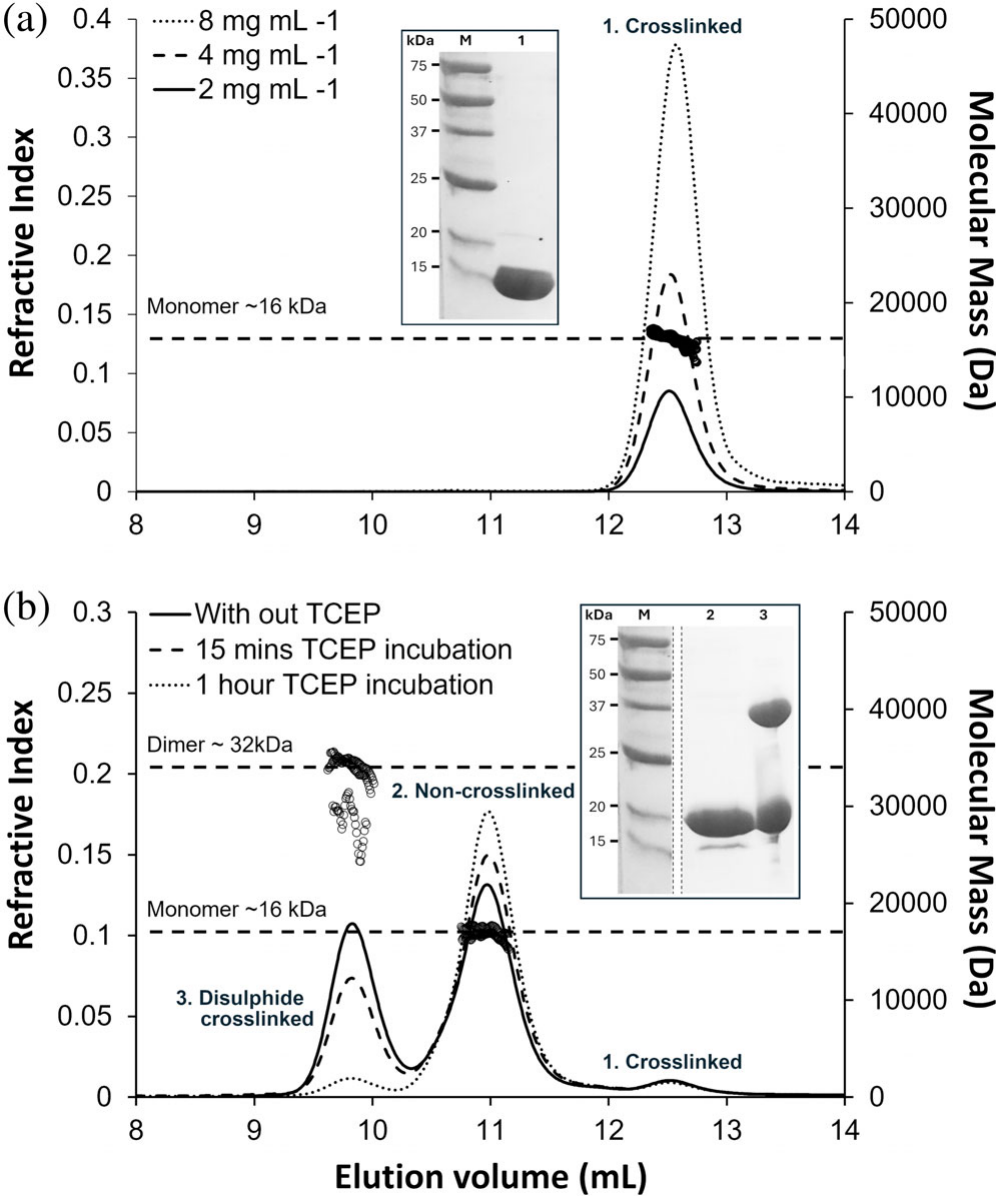
Characterization of purified WT Cpe0147 and T450C mutant by SEC‐MALLS and SDS‐PAGE. (a) SEC‐MALLS trace of Cpe0147^439‐587^ at three different concentrations shows a single crosslinked species eluting at 12.5 mL and with a diagnostic mass of 16 kDa. This is consistent with the reducing SDS‐PAGE analysis (inset). (b) SEC‐MALLS trace of T450C mutant (5 mg mL^−1^) incubated with 2 mM TCEP for 0, 15, and 60 min as labeled. The trace displays three distinct peaks, from left to right, a disulfide crosslinked dimer (mass 32 kDa; elution vol. 9.8 mL), a non‐crosslinked monomer (mass 16 kDa; elution vol. 11 mL), and crosslinked monomer (mass 16 kDa; elution vol. 12.5 mL). The result is consistent with SDS‐PAGE analysis (inset) where a reducing environment shows 97% non‐crosslinked and 3% crosslinked species, and a non‐reducing has an additional disulfide crosslinked dimer with apparent mass of 37 kDa. Parts of the gel images have been removed for clarity.

The hydrodynamic properties of wild type and T450C species in solution were investigated using SEC‐MALLS. This technique has the advantage of separating mass calculation from shape or dynamic characteristics of the protein in solution. The wild‐type protein displays a single species eluting at 12.5 mL with a calculated mass of ~16 kDa as expected for a monomeric species (Figure 3a). By contrast, a more complex pattern of three elution peaks is produced by the T450C mutant with peaks at 9.7, 11.0, and 12.5 mL (Figure 3b). In agreement with SDS‐PAGE, the largest species, eluting at 9.7 mL with a mass of 32 kDa, is a disulfide crosslinked dimer, followed by a non‐crosslinked species eluting at 11 mL with a monomeric mass of 16 kDa (Figure 3b). In this system, by adding increasing concentrations of the reducing agent (TCEP), conversion of S‐S crosslinked dimer to non‐crosslinked monomeric protein is observed, again consistent with SDS‐PAGE under reducing and non‐reducing conditions. Only a very small proportion of T450C protein forms the smallest hydrodynamic radius, crosslinked species eluting similarly to the wild‐type protein at ~12.5 mL and with a thioester crosslink confirmed by mass spectrometry (Figure S3).

### 2.3 The X‐ray structure of the T450C mutant shows an unreactive nucleophile and provides a ground state model in a serine protease‐like mechanism

**FIGURE 4:**
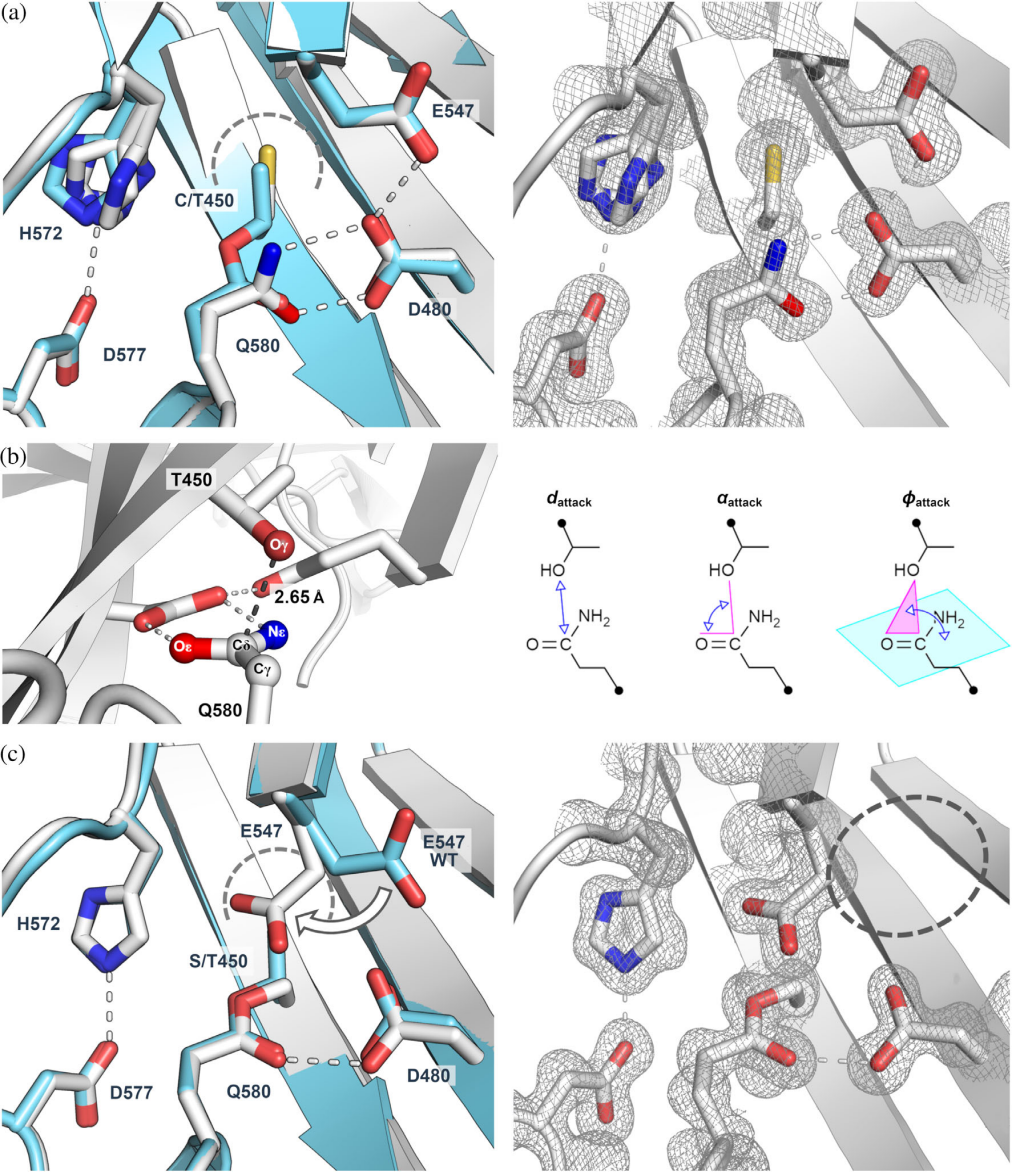
Crystal structures of Cpe0147^439‐587^ T450C and T450S mutants support a serine protease‐like mechanism of ester bond formation. (a) Structural overlay of non‐crosslinked T450C structure (PDB ID 9BLO; white cartoon and stick models) and the crosslinked WT structure (PDB ID 4NI6; aquamarine). The cysteine thiol (gold) occupies the same location (indicated by a dashed curved) as the WT threonine methyl group, and the lack of a crosslink allows Q580 to form a bidentate hydrogen bond to the buried acid D480. The T450C histidine base, H572, adopts three conformations, one of which can hydrogen bond to its D577 partner. (b) A ground state model of the WT crosslinking site modeled on the T450C structure. T450 Oγ closely approaches Q580 Cδ. The electrophile is held flat by hydrogen bonding to the buried acid and in a geometric arrangement consistent with that of serine proteases. Geometric parameters *d*_attack_, *α*_attack_, and *φ*_attack_ are defined in the same way as Du et al. (Du et al. 2025). (c) Structural overlay of the crosslink site of the T450S mutant (PDB ID 9BLP; white cartoon and stick model) and the WT protein (PDB ID 4NI6; aquamarine). The T450S mutation allows the E547 side chain to rotate towards the ester bond to fill space (indicated by a dashed curve) not occupied by a WT T450 methyl group. The E547 carboxylate/carboxylic acid functionality sits directly above the ester bond. Electron density maps (2fo‐fc maps at 1.0 RMSD contouring) in (a, b) show the highlighted crosslinking (or lack thereof), H572 conformational flexibility, and E547 side localization unambiguously.

The crystal structure of T450C Cpe0147^439‐587^ (PDB ID 9BLO) was determined at 1.3 Å resolution with two molecules in the asymmetric unit (Table S2). The two molecules are near‐identical to the wild‐type structure, with RMSD values of 0.53 and 0.44 Å, respectively, for Cα atoms of chain A and chain B of the mutant when compared to wild type (PDB ID 4NI6). A close inspection of the mutation site shows no thioester crosslink with the C450 thiol group sequestered in the pocket otherwise occupied by the methyl group of the wild‐type T450 (Figure 4a). The reactive sidechain of Q580 forms hydrogen bonds through both Oε1 and Nε2 atoms with the protonated buried acid, D480. The position of E547 is unchanged compared to the wild type, pointing away from the ester bond position and making a hydrogen bond with D480.

A wild‐type, non‐crosslinked model was produced from the T450C structure (Figure 4b). Geometric analysis following the method of Du et al. formulated for serine protease characterization gives an attack distance, *d*_attack_, of 2.65 Å measured between nucleophile Oγ and electrophile target atom Cδ (Du et al. 2025). The attack angles *α*_attack_ and *φ*_attack_ are calculated at 104° and 89°, respectively. Comparative average values for these parameters calculated from 1000+ serine protease crystal structures are *d*_attack_ = 2.68 (SD = 0.14 Å), *α*_attack_ = 93° (SD = 7°), and *φ*_attack_ = 84° (SD = 8°).

### 2.4 The T450S mutant X‐ray structure shows a rearrangement of one of the proposed oxyanion‐like buried acids

The crystal structure of T450S Cpe0147^439‐587^ (PDB ID 9BLP) was determined at 1.2 Å (Table S2) with one molecule in the asymmetric unit. The T450S protein, similarly to T450C, closely resembles the wild type with an RMSD comparison of 0.66 Å across all Cα atoms. An intramolecular ester bond linking S450 and Q580 is unambiguously present (Figure 4c). The pocket occupied by the T450 methyl group in the wild type or thiol in the T450C mutant is partly occupied by the buried acid E547, which, having rotated outwards from the wild‐type localization, no longer forms a proton shuttle with D480 and the ester bond carbonyl. Instead, E547 hydrogen bonds to an adjacent backbone carbonyl of V110 and stacks directly above the ester bond ether oxygen, making a close contact (E547 Oε1–S450 Oγ, 3.2 Å).

### 2.5 Molecular simulations explain the differences in hydrolysis seen for the S450 mutant and support a serine protease‐like mechanism

We performed equilibrium molecular dynamics (MD) and metadynamics simulations of WT Cpe0147^439‐587^ and a set of the experimentally tested mutants to further tease apart the role of putative catalytic triad residues and the susceptibility of the ester bond to hydrolysis when a serine nucleophile replaces threonine.

To recapitulate the proposed serine protease‐like mechanism of Cpe0147^439‐587^, we first simulated the D577H mutant to assess how D577 interacting with H572 would facilitate the correct positioning of H572 to act as a base for the proton abstraction of T450. In both WT and D577H proteins, the Nδ atom of H572 and the hydroxyl proton of T450 approach closely enough to effect protein abstraction (Figure S4a,b). Similarly, T450 is found in close proximity and in suitable geometries to form the ester bond with Q580. In both mutants, the dynamic behavior of H572 relative to T450, and T450 relative to Q580, is near identical, with the distance distributions predominantly overlapping in both systems (Figure S4c,d) and consistent with bond formation (Figure 2).

Additionally, we simulated the T450S/H572E double mutant to assess if glutamate can act as an acid/base in our mechanism. In line with the experimental bond formation efficiency (100% (WT) vs. 87% crosslinked (T450S/H572E); Figure 2), the distribution of distances between E572 and S450, and between H572 and S450 overlap, with E572 even more closely approaching the nucleophile proton (Figure S5). A shift in the rotameric behavior of E572 relative to the WT histidine (compare Figure S4h with Figure S5h) shows the E572 sidechain often pointing towards the solvent, away from T/S450 (Figure S5g). This would overall suggest a reduced catalytic efficiency of T450S/H572E, as observed experimentally.

**FIGURE 5:**
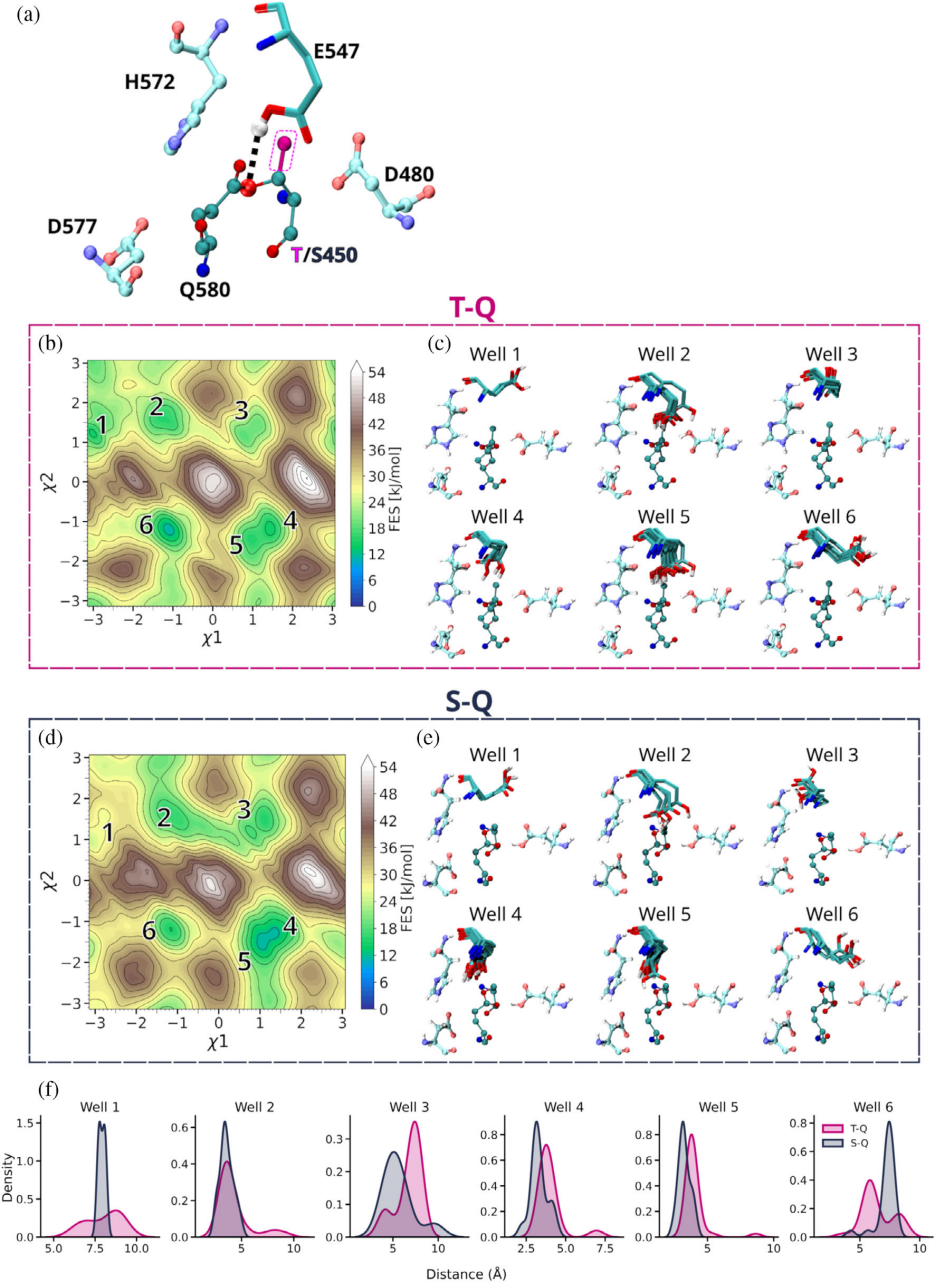
Simulations suggest why the T450S S‐Q ester bond is more readily hydrolyzed compared to a WT T‐Q bond. (a) Representative conformation of the T‐Q covalent bonding and surrounding residues found in Cpe0147. E547 is highlighted for its ability to form a hydrogen bond with the ether oxygen of the ester bond in WT T450 protein (dashed black line and the E547 side chain proton shown as a white sphere). The T450 methyl group is highlighted in magenta. (b, d) Two‐dimensional free energy surfaces (FES) of the Thr‐Gln or Ser‐Gln simulated constructs, as obtained from metadynamics simulations using, as collective variables, the E547 χ1 and χ2 dihedral angles. (c, e) Conformational ensembles of E547 isolated from the energy minima identified within the reconstructed FES (1 to 6 in panels (b) and (d)). (f) Probability density distributions of the E547:HE1‐T/S450:OG1 distance shown in (a) and obtained for each of the six identified energy minima.

In a potential hydrolysis mechanism, the residue acting as acid to donate a proton back to the S450 nucleophile is unclear. The positioning of H572 in our crystals and comparison to a classic serine protease hydrolysis mechanism suggest that this histidine could act as a catalytic acid to protonate the ester bond serine. However, our simulations suggest the key player in hydrolysis to be E547. In the WT structure, the carboxylic group of E547 is buried within the protein interior away from the ester bond and is likely protonated and H‐bonded to the second buried acid, D480. The T450S structure instead shows the E547 side chain rotated out of the protein interior and stacking directly above the ester bond ether's oxygen (Ser‐450 Oγ). By biasing E547 χ_1_ and χ_2_ dihedrals, metadynamics revealed the thermodynamics underlying the conformational space of E547, showing markedly different conformational energies between WT and T450S mutant. The free energy surface of WT Cpe0147^439‐587^ features six low‐energy conformations, listed 1–6 in Figure 5b. Three conformations support hydrolysis (2, 4, 5) with E547 stacked above the ester bond, and three (1, 3, 6) have the side chain pointing away from the ester bond, unable to effect hydrolysis (Figure 5c). Well 6 has the lowest energy, preserving the WT H‐bond with E480, and shows an energetic barrier of at least 28 kJ mol^−1^ relative to hydrolysis‐promoting basins (Figure 5c). T → S substitution (Figure 5d,e) enhances the ability of E547 to swing towards the ester bond to facilitate hydrolysis. In this case, E547 is unlikely to sample non‐hydrolyzing Well 1, and the barrier between hydrolyzing Well 2 and non‐hydrolyzing Well 3 is noticeably lowered (~20 kJ mol^−1^) and unlikely hinders conformational switching. In T450S, the most likely conformations are represented by hydrolysis‐promoting Basins 4 and 5, with E547 directly stacking above the ester bond. The differences between the threonine and serine‐containing systems are evident in the distribution of distances between reactive groups in the different wells. For Wells 4 and 5, the mean distance between E547 HE1 and S450 Oγ falls at approximately 3.5 Å, in line with what is observed in the crystal structure of this mutant. Conversely, the WT threonine offsets the same distance to higher values (Figure 5f).

Distance distributions, p*K*_a_, and side chain burial were calculated across the simulated trajectories for WT and T450S for consistency with an enzyme‐like mechanism (Figures S6 and S7). Distances indicating a network of hydrogen bonding can be seen between several residues (Figure S6), including putative base (H572) and nucleophile (T/S450), buried acids D480 and E547, and between the putative electrophile (Q580) side chain carbonyl and a protonated D480 (Figure S6d). The Q580 electrophile side chain amine appears to transiently associate with the buried acid D480 (Figure S6e), while the base–acid pairing (H572‐D557) is mediated in the WT by weak hydrogen bonding. The interaction networks are overall consistent with D480 and E547 being protonated and buried as indicated by the p*K*_a_ value distributions illustrated within Figure S7.

## 3 DISCUSSION

**FIGURE 6:**
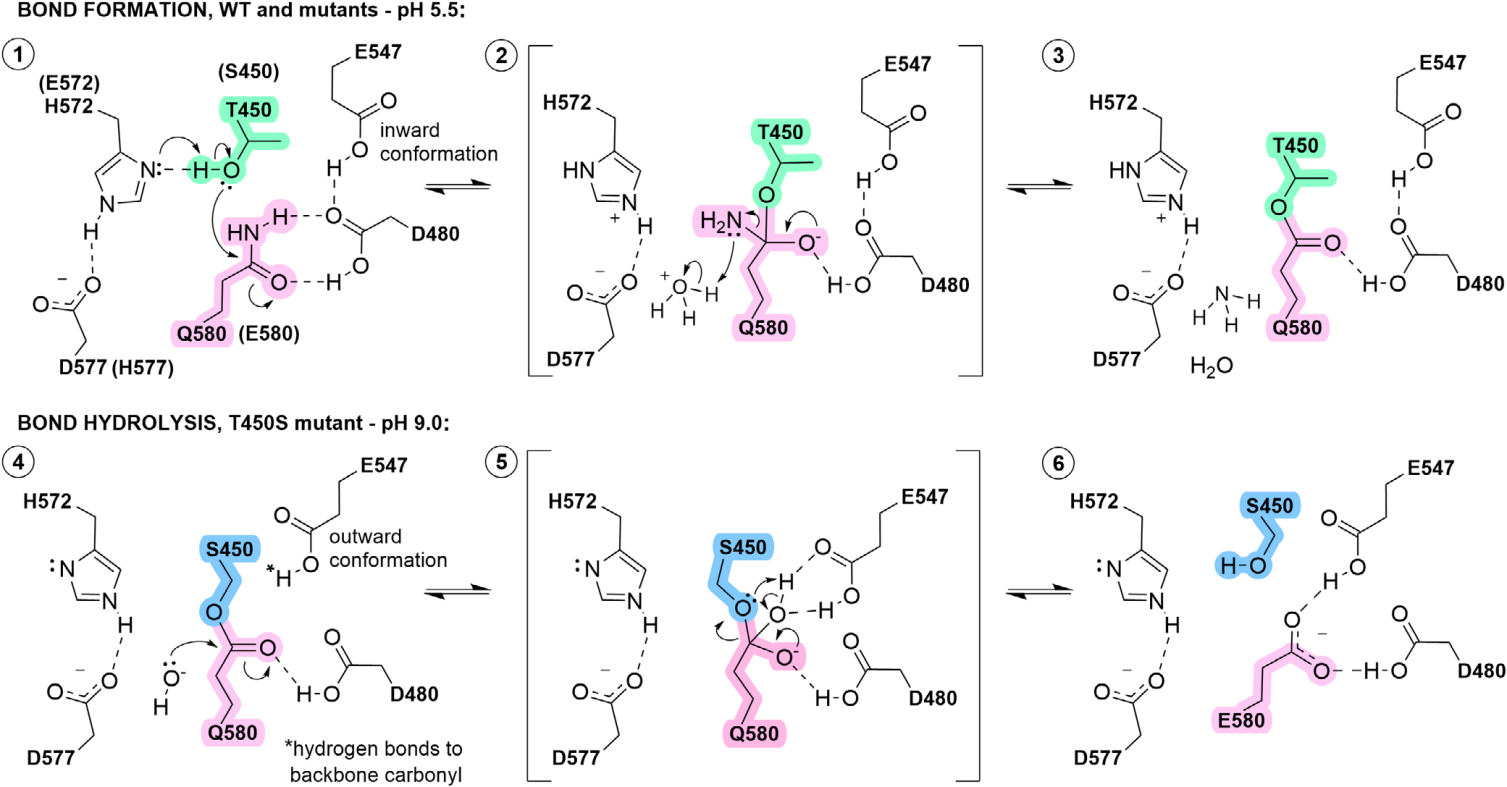
A refined mechanism of intramolecular ester bond formation and hydrolysis in bacterial adhesin domains. Panel 1: a wild‐type model showing the position of the active site residues prior to ester bond formation was derived from the non‐crosslinked T450C crystal structure (PDB ID 9BLO). One of the H572 rotamers derived from the T450C structure would be in hydrogen bonding distance to the WT T450 Oγ1 oxygen ready for proton abstraction. The bond forming E580 forms a bifurcated hydrogen bond to the buried acid residue D480. The Q580 Cδ atom is presented at an appropriate distance and geometry for nucleophilic attack by the T450 hydroxyl group. Panels 2 and 3: a proposed tetrahedral intermediate state formed under acidic conditions collapses to form the final crosslinked structure (PDB ID 4NI6) with the loss of ammonia. Panel 4: at pH 9, the S450 mutant undergoes attack by a hydroxide ion in solution. This configuration is modeled on the T450S crystal structure (PDB ID 9BLP) with E547 in an outward conformation. Panels 5 and 6: a tetrahedral intermediate is stabilized by both buried acids directly prior to its collapse. Proton abstraction from E547 regenerates the active site with E580 in place of the original Q580. Note that multiple steps, and electron and proton movements are combined and simplified; transition states are not shown. The revised mechanism is derived from that of Kwon et al. (2014) and is supported by X‐ray crystal structures and MD simulations. Drawn using ChemDraw version 23.1.2 (Revvity Signals Software Inc.).

Adhesins in Gram‐positive bacteria have evolved as single‐molecule wide constructions featuring intramolecular covalent crosslinks that have markedly changed our views on protein stability (Kang et al. 2007; Kang et al. 2009; Kwon et al. 2014). We have proposed that the intramolecular ester bond crosslinking present in a subset of these adhesins represents a trapped acyl intermediate equivalent to that of a serine protease mechanism prior to hydrolysis (Radisky et al. 2006). We have herein detailed structural, chemical, and dynamics factors determining ester bond formation in the *Clostridium perfringens* adhesin Cpe0147, and confirm a serine protease‐like mechanism involving a catalytic triad (acid/base/nucleophile) or a dyad (base/nucleophile) and the equivalent of an oxyanion hole, as summarized in Figure 6.

Perhaps the most compelling evidence for convergent evolution is the ground state configuration of WT protein derived from the T450C crystal structure (Figures 4b and 6, panel 1). In this model, the close approach (2.65 Å) of nucleophile threonine Oγ and electrophile glutamine Cδ, and the perpendicular relative orientation of the side chains, are consistent with the geometric arrangement observed by Du et al. within 1000+ serine proteases (Du et al. 2025). This close approach effectively destabilizes the ground state of the system.

Du et al. further apportion the relative contribution of other elements towards the overall catalytic efficiency in serine proteases including the dominance of the catalytic base, contributing as much as half of the overall catalytic impetus, and the non‐essential requirement of a catalytic acid (Du et al. 2025). Similarly, in our system, the inclusion of either a histidine or glutamate (or aspartate to a lesser extent) as a catalytic base appears essential. A catalytic dyad (minus the catalytic acid) appears equally viable given the D577A mutant retains 86% of the WT esterification. The D577H mutation that recapitulates the catalytic machinery of the cytomegalovirus protease removes the putative acid but retains a potential hydrogen bond donor atom. Studies by Khayat et al. suggested that the cytomegalovirus protease is likely to function via a catalytic dyad, but less efficiently compared to other serine proteases utilizing triads, and this correlates with our system (Khayat et al. 2001).

The recovery of T450S activity from 51% crosslinking efficiency to 87% by the addition of the second mutation, H572E, highlights the importance of a suitable base functionality in the putative catalytic dyad/triad. The T450S/H572E protein recapitulates the catalytic triad machinery of the serine protease sedolisin that is secreted by *Pseudomonas* species and which can hydrolyze insulin. Sedolisin is active between pH 3.0 and 5.0, bracketing the standard p*K*_a_ of a glutamic acid side chain of ~4.1, allowing this side chain to function as acid or base as required by different steps in catalysis (Reichard et al. 2006; Wlodawer et al. 2001; Wlodawer et al. 2003). While we did not test the pH dependence of our double mutant, our standard catalytic assay is completed at a relatively low pH of 5.5, close to the functional range of sedolisin. In search of a sedolisin‐like glutamate base in nature, we identified more than 400 UniProt entries of putative bacterial adhesins containing predicted structures of ester bond crosslink domains. While crosslinks cannot be predicted by AlphaFold, the conserved protein topology, overall amino acid sequence similarity, and the proximity and relative orientation of viable acid/base/nucleophile/electrophile and oxyanion‐like hole residues allowed us to confidently assign domains as homologous to the canonical Cpe0147 repeat domain and its catalytic machinery. Of note, a glutamate variation does appear within nature; however, this was found in less than 0.5% of the UniProt entries surveyed. The two exemplars of the glutamate base in domains from *Agathobacter rectalis* and an unclassified *Lachnospiraceae* bacterium also display a paired asparagine as “acid” and further support the possibility of a functional catalytic dyad operating rather than a triad. The crosslinking potential of these putative adhesin domains remains to be validated experimentally.

In our experiments, the bond forming nucleophile/electrophile residues appear restricted to combinations of threonine/glutamine, threonine/glutamate, serine/glutamine, and serine/glutamate. While still catalytically viable, even conservative substitutions, for example, Q580E in the threonine/glutamate combination, greatly diminish bond formation, in this case to 35% of the wild‐type yield. The differences we observe between the crosslinking ability of the two well‐characterized nucleophiles cysteine and threonine suggest a finely regulated crosslinking propensity, strongly dependent on the micro‐environment of the reactive species. Cysteine is an obvious nucleophile involved in many biological reactions, including the cleavage of peptide bonds by cysteine proteases (Otto and Schirmeister 1997), the formation of thioester bonds in complement proteins (Pangburn 1992), and the attachment of a raft of Gram‐positive bacteria including *Clostridium perfringens* and *Streptococcus pyogenes* to host cells (Linke‐Winnebeck et al. 2014; Miller et al. 2018; Walden et al. 2015). However, a cysteine nucleophile in our system disfavors intramolecular crosslinking as the thiol group, already compromised by lower polarity than the WT hydroxyl, is also buried within the protein. A similar sequestration of the thiol group was noted in other serine protease‐like enzymes where a catalytic threonine was replaced by a cysteine (Reichard et al. 2006; Wlodawer et al. 2003). The highly dynamic solution properties of the non‐crosslinked protein as indicated by SEC‐MALLS and previously by NMR experiments (Young et al. 2017), likely contribute to this lack of reactivity.

Our non‐exhaustive survey of putative adhesin domains in the UniProt Database suggests that the vast majority of ester bond‐forming sites in adhesin domains have evolved with a threonine as nucleophile, placing the methyl group in a pocket adjacent to the conformationally restricted E547 and likely minimizing the potential for ester bond hydrolysis. Our simulations highlight a large energy barrier to E547 rotation. The methyl group of T450 closely interacts with the acyl chain of E547 and a large desolvation energy likely regulates the ability of E547 to rotate and attack the ester bond, as we see for the T450S system. However, in our adhesin domain‐based molecular superglues from *Gemella bergeri* and *Enterococcus columbae* used extensively in our laboratory (Young and Squire 2020), a serine nucleophile does not promote hydrolysis even at pH 10 and when heated to 37–60°C (*unpublished work*). Additionally, a naturally occurring serine nucleophile‐containing domain is predicted in the super‐sized adhesin from *M. mulieris*, a bacterium naturally found in an acidic natural environment of pH 3–5 (Young et al. 2023). Assuming this native domain follows the pattern of the laboratory superglues in forming stable ester bond crosslinks, the autocatalytic site configuration in these systems is more complex than a simple substitution of a single amino acid in isolation, with the sites likely co‐evolving in shape and chemistry to negate the hydrolysis potential.

Mechanistically, the ground state of bond formation emulates the active site configuration within the non‐crosslinked C450 crystal structure and shows how the histidine or glutamate base deprotonates the T450 or S450 nucleophile (Figure 6, panel 1). MD simulations of H572 are characterized by subsets of well‐defined conformers, some showing the Nδ of the histidine imidazolium ring approaching the T450 Oγ1 oxygen within distances and geometries optimal for abstraction (Figure S6a). The role of the buried and protonated D480 as an oxyanion hole equivalent is crucial for the bond formation, and following Du et al., we suggest its interactions with Q580 destabilize the ground state (Figures S6d,e and S7) (Du et al. 2025). Replacing the D480 aspartic acid with an asparagine abolishes activity, both disrupting hydrogen bonding and removing a polarizing potential to the Q580 carbonyl bond. Stabilization of the putative oxyanion intermediate would also be ablated by this substitution. In the proposed intermediate state (Figure 6, panel 2), we suggest the involvement of a hydronium ion donating a proton to the leaving group, rather than the histidine that would play this role in a standard serine protease mechanism. This presumes that H572 is sequestered, as seen in the crystal structures of crosslinked WT and T450S domains, and is no longer in a suitable location and orientation for proton donation. Our experiments show that ester bond formation favors a pH <7.0 (Young et al. 2017; Young and Squire 2020) where hydronium ions are present in excess, and this further supports our acid‐mediated mechanism.

With respect to the mechanism of hydrolysis, as shown in the T450S crystal structure and reiterated by metadynamics simulations, the presence of the smaller serine relative to threonine considerably increases the likelihood of E547 directing its carboxyl group outward and close to the ether oxygen of the ester bond. At the higher pH values that promote hydrolysis, we propose that a hydroxide ion attacks the Q580 carbonyl to form an oxyanion intermediate stabilized by the outward facing, but still buried and presumably protonated E547 side chain (Figure 6, panels 4 and 5). The interactions formed with E547 would orient the newly acquired hydroxyl functionality, facilitating the abstraction of the hydroxyl proton by the serine nucleophile as the tetrahedral intermediate collapses. The final state depicted in Figure 6, panel 6, represents a catalytic ground state of the serine protein ready for (re‐)crosslinking if the pH is dropped to 5.5, but features a glutamate side chain in a hydrogen bond configuration different from that of the glutamine in panel 1. This altered and less perpendicular arrangement relative to the serine nucleophile results in less efficient bond formation as observed for the Q580E mutant at 35% of WT efficiency (Figure 2).

Our findings reveal more of the intricate details behind ester bond formation within adhesin repeat domains that illustrate how evolutionary pressures (DNA change, selection, and functional optimization) can drive distinct lineages towards the same biochemical solutions. A catalytic triad remarkably similar in geometric configuration to serine proteases most efficiently forms a crosslink. But our recreation of functional human cytomegalovirus protease‐like machinery shows that a catalytic dyad can also drive esterification. Given the flexibility in catalytic residue choice seen across all serine proteases, it is unsurprising that more than one configuration of this system is also catalytically viable (Du et al. 2025; Ekici et al. 2008; Polgar 2005). Like serine proteases, we see in crystal structures/simulations and propose in our refined catalytic mechanism that coordinated motion of base, nucleophile, and electrophile promotes catalysis (Radisky et al. 2006). These findings provide a compelling argument for convergent evolution in bacterial adhesin domains having “reimagined” serine protease machinery with a twist—by making bonds rather than breaking them.

## 4 MATERIALS AND METHODS

### 4.1 Cloning and site directed mutagenesis of WT and mutants of Cpe0147 (aa 439–587)

Constructs encoding Cpe0147^439‐587^ (WT and mutant sequences derived from UniProt A0AAV3BQK0) were cloned into a modified pProExHta (Invitrogen) vector (pMBP‐ProExHta), using EcoRI and KasI restriction endonucleases and T7 DNA ligase. pMBP‐ProExHta was generated by inserting the *E. coli* maltose binding protein (MBP) gene between the His_6_‐tag and the recombinant tobacco etch virus (TEV) protease (rTEV) cleavage site of pProExHta (Ting et al. 2015). Site‐directed mutagenesis was carried out using a whole plasmid PCR method with complementary mutagenic primers (Dominy and Andrews 2003), iProof enzyme (BioRad), DpnI treatment, and T7 DNA ligase for re‐circularization before transformation into *E. coli* DH5α. Correct in‐frame cloning and mutagenesis was confirmed by Sanger sequencing provided by the Auckland Genomics Centre, The University of Auckland, Auckland, New Zealand.

### 4.2 Expression and purification of WT and mutants of Cpe0147 (aa 439–587)

All Cpe0147^439‐587^ WT and mutant constructs were transformed into *Escherichia coli BL*21 (DE3) cells and expressed at 18°C overnight in Terrific Broth medium supplemented with 50 mg mL^−1^ ampicillin and with shaking at 200 rpm. Cells were harvested by centrifugation, mechanically lysed, and subjected to immobilized metal affinity chromatography (IMAC), rTEV cleavage, and reverse IMAC treatments, as described previously (Young et al. 2017). Proteins were concentrated to 1 mL in volume and purified by size‐exclusion chromatography (SEC) using a Superdex 75 16/60 column equilibrated with SEC buffer (20 mM Tris HCl pH 8.0, 100 mM NaCl and 2 mM βME). Fractions containing the target proteins were concentrated to 100 mg mL^−1^ and characterized by SDS‐PAGE. In mixed samples of crosslinked and non‐crosslinked protein, the species were separated by their size/hydrodynamic radius relative to elution volume, and were separately flash cooled in liquid nitrogen for later use.

The presence or absence of an intramolecular ester bond crosslink in the protein domains was assessed by SDS‐PAGE immediately following purification. SDS‐PAGE band intensity was quantified using ImageJ (Schneider et al. 2012) and the presence of crosslinking was confirmed by mass spectrometry. LC–MS/MS characterization was performed as a service by the Mass Spectrometry Centre, The University of Auckland, Auckland, New Zealand.

### 4.3 Protein crystallization, data collection, and refinement

The non‐crosslinked T450C Cpe0147^439‐587^ was concentrated to 150 mg mL^−1^ and crystallized using sitting drop vapor diffusion using the Oryx 4 robot (Douglas Instruments) by mixing 200 nL protein with 200 nL of crystallization solution comprising 0.2M MgCl_2_·6H_2_O, 0.1M Tris·HCl pH 8.5, and 30% (w/v) PEG 4000. The crosslinked T450S mutant Cpe0147^439‐587^ was concentrated to 350 mg mL^−1^ and crystallized similarly from a solution comprising 0.2M sodium thiocyanate and 20% PEG 3350. Both crystals were cryoprotected (crystallization solution supplemented with 20% (v/v) glycerol), mounted in nylon loops, and flash cooled in liquid nitrogen.

X‐ray diffraction data were collected at the Australian Synchrotron using the Blu‐Ice software on the MX1 beamline, and were processed using XDS and AIMLESS (Carter and Wells 1988; Evans and Murshudov 2013; Kabsch 2010; McPhillips et al. 2002). The structures were determined by molecular replacement in PHASER (Hedstrom 2002) using the structure of the wild‐type protein (PDB ID 4NI6) (Kwon et al. 2014; McCoy et al. 2007). Iterative model building and refinement, including the addition of water and ions, used COOT and REFMAC (Emsley and Cowtan 2004; Murshudov et al. 2011). Coordinates and structure factors were deposited in the Protein Data Bank (Berman et al. 2003) with identifiers 9BLO and 9BLP for T450C and T450S structures, respectively. Data collection and refinement statistics are given in Table S2. A wild‐type ground state model of the crosslinking site was produced from the T450C structure by mutating the cysteine to a threonine using COOT and then energy minimizing the coordinates. Geometric parameters *d*_attack_, *α*_attack_, and *φ*_attack_ were calculated from selected minimized atomic coordinates following the methodology of Du et al. (Du et al. 2025).

### 4.4 Size‐exclusion chromatography coupled to multi‐angle laser light scattering

Size‐exclusion chromatography coupled to multi‐angle laser light scattering (SEC‐MALLS) analysis was used to determine the molecular mass, crosslink presence/absence, and hydrodynamic properties of the proteins in solution. Protein (100 μL at 2–10 mg mL^−1^) was loaded onto a Superdex 75 10/300 GL column equilibrated in 10 mM Tris HCl pH 8.0, 150 mM NaCl, 3 mM NaN_3_, mounted on a Dionex HPLC with a PSS SLD7000 7‐angle MALLS detector and Shodex RI‐101 differential refractive index detector. The PSS winGPC Unicorn software determined averaged molecular masses for the eluted protein species.

### 4.5 Molecular dynamics and metadynamics simulations

Simulations were performed using Gromacs 2021.7. The protein was protonated at pH 7.0 and placed in a box measuring 20 × 12 × 12 nm, solvated with TIP3P water molecules. Na^+^ and Cl^−^ ions were added to achieve system neutrality to a total ionic strength of 0.15M. Particles were assigned parameters from the Amber ff14SB force field (Maier et al. 2015). The system was first energy minimized to optimize atomic positioning, using a steep descent algorithm for 50,000 steps with an energy step of 0.01 nm and a tolerance of 10 kJ mol^−1^ nm^−1^. Solvent equilibration was achieved in two steps: first, a 1‐ns‐long simulation was performed in the NVT ensemble with temperature kept constant at 300 K, coupled every 2.0 fs using a V‐rescale thermostat (Berendsen et al. 1984). In this step, each atom was assigned random velocities, according to a Maxwell‐Boltzmann distribution obtained at the simulated temperature. A second equilibration step was performed in the NPT ensemble, keeping both temperature and pressure constant at 300 K and 1.0 bar, respectively. Pressure was coupled every 2.0 fs using the Parrinello‐Rahman barostat (Parrinello and Rahman 1981). During both equilibration steps, protein atoms were positionally restrained, applying a force constant of 1000 kJ mol^−1^ nm^−2^. A 500‐ns‐long run was then performed without positional restraints in the NPT ensemble.

Metadynamics simulations were performed using the last conformer of the equilibration run as the starting conformation. For each system (wild type and T450S mutant) three metadynamics simulation replicates were performed until convergence, for a total simulated time of 6.0 μs per run (Table S3). The replicates differed in the set of initial velocities generated in the NVT equilibration step, as described above, and in the history of the bias added to explore the phase space along the chosen collective variables (χ_1_ and χ_2_ dihedral space). The rotameric space of the χ_1_ and χ_2_ dihedrals of residue 547 was explored and biased every ps by adding Gaussians of energy 0.2 kJ mol^−1^ high and 0.2° wide. The free energy profiles from the metadynamics simulations were then obtained by reweighing each replicate to discount the bias accumulated during the simulations, according to the protocol described by Tiwary and Parrinello (Tiwary and Parrinello 2015). Convergence was assessed for each replicate by computing the difference in the estimated free energy for each of the populations sampled by the χ_1_ and χ_2_ dihedral angles (Figure S8). Errors on the free energy surfaces were estimated via block error analysis (Figures S9 and S10), using a block size of 1000, as at this block size the variance of the mean had reached a plateau.

A second set of molecular dynamics simulations, at equilibrium, was performed on WT Cpe0147 (Kwon et al. 2014), T450S (Young et al. 2017), D577H, and T450S/H572E mutants, using a simulation protocol for box creation, solvation, and equilibration identical to that described above. Following equilibration, each system was simulated five times for 250 ns each (total simulated time 1.25 μs), with each replicate differing for the set of initial velocities assigned in the NVT equilibration step. The first 50 ns of each replica were discarded from analysis, as considered equilibration time. Protonation states were set to those occurring at pH 7.0, except for D480, E547, H572, and D577. D480 and E547 remained protonated for both mutants, while H572 and H577 in the D577H mutant were both singly protonated on the Nε atom. Residue E572 in the T450S/H572E mutant was left deprotonated. A summary of all the simulated systems is provided in Table S3. Unit root test statistics informing on the convergence of the monitored observables are provided in Table S4, together with the critical values expected at different confidence levels (MacKinnon 2010).

All analyses were performed using a combination of *in‐house* scripts on the collected trajectories and MDAnalysis (Gowers et al. 2019). Representative structures were obtained using VMD 1.9.4 (Michaud‐Agrawal et al. 2011). p*K*_a_ and percentage buried values were computed using PROPKA3 (Olsson et al. 2011; Søndergaard et al. 2011).

### 4.6 Bioinformatics and predicted structure analysis

A keyword search of the UniProt database (search terms; “T‐Q ester bond,” “VafE repeat”) found more than 1500 protein entries. For those with AlphaFold predicted structures, ester bond crosslink domains and putative autocatalytic amino acids were identified by visual inspection using PyMol (Schrodinger) or Coot graphics software and by comparison to canonical ester bond domain Cpe0147 (PDB ID 4NI6) (Emsley and Cowtan 2004).

## AUTHOR CONTRIBUTIONS

**Yuliana Yosaatmadja:** Conceptualization; investigation; methodology; visualization; writing – original draft; writing – review and editing. **Vanessa Ung:** Investigation; formal analysis; visualization; writing – review and editing. **Xinlu Liu:** Investigation. **Yixuan Zhao:** Investigation. **Julia K. Wardega:** Investigation. **Aria Shetty:** Investigation. **Sophie Schoensee:** Investigation. **Ivanhoe K. H. Leung:** Conceptualization; funding acquisition; writing – review and editing. **Jeremy R. Keown:** Investigation; methodology; writing – review and editing. **David C. Goldstone:** Methodology; writing – review and editing. **Edward N. Baker:** Conceptualization; funding acquisition; writing – review and editing; supervision. **Paul G. Young:** Conceptualization; funding acquisition; methodology; writing – review and editing; supervision. **Davide Mercadante:** Conceptualization; methodology; formal analysis; writing – review and editing; supervision. **Christopher J. Squire:** Conceptualization; funding acquisition; investigation; methodology; visualization; writing – original draft; writing – review and editing; supervision; project administration.

## Supporting information


**Table S1.** Structural and sequence comparison of ester bond adhesin domains released in the Protein Data Bank.
**Table S2.** Data collection, refinement, and validation statistics for T450C and T450S X‐ray crystal structures.
**Table S3.** Molecular dynamics and metadynamics replicate and sampling parameters.
**Table S4.** Unit‐root test statistics for time traces of the distances between mechanistically relevant residues.
**Figure S1.** The active site triads of two non‐cannonical serine proteases.
**Figure S2.** Naturally occurring variations in the putative catalytic residues of Cpe0147‐like ester crosslink domains.
**Figure S3.** Trypsin digest coupled with tandem mass spectrometry for the T450C mutant.
**Figure S4.** Metadynamics simulations of stabilizing interactions for T‐Q bond formation in Cpe0147.
**Figure S5.** Metadynamics simulations of stabilizing interactions for S‐Q bond formation in Cpe0147.
**Figure S6.** Distance distributions of the interactions made by catalytically relevant residues in molecular dynamics simulations.
**Figure S7.** Distributions of p*K*_a_ and percentage buried for catalytically relevant residues in molecular dynamics simulations.
**Figure S8.** Convergence of the free energy values along χ_1_ and χ_2_ in metadynamics simulations.
**Figure S9.** Block error analysis of free energy across collective variables in metadynamics simulations.
**Figure S10.** Errors associated with free energy surfaces in metadynamics simulations.

## Data Availability

The data that support the findings of this study are available from the corresponding author upon reasonable request.
