# Supplementary material for: Protease mimicry: Dissecting the ester bond crosslinking mechanics in bacterial adhesin proteins
Source: Protein Sci. 2025 Jul 26;34(8):e70238. doi: 10.1002/pro.70238 (PMC12679949; doi:10.1002/pro.70238)
Supplement: Supplementary file 1 — Table S1. Structural and sequence comparison of ester bond adhesin domains released in the Protein Data Bank. Table S2. Data collection, refinement, and validation statistics for T450C and T450S X‐ray crystal structures. Table S3. Molecular dynamics and metadynamics replicate and sampling parameters. Table S4. Unit‐root test statistics for time traces of the distances between mechanistically relevant residues. Figure S1. The active site triads of two non‐cannonical serine proteases. Figure S2. Naturally occurring variations in the putative catalytic residues of Cpe0147‐like ester crosslink domains. Figure S3. Trypsin digest coupled with tandem mass spectrometry for the T450C mutant. Figure S4. Metadynamics simulations of stabilizing interactions for T‐Q bond formation in Cpe0147. Figure S5. Metadynamics simulations of stabilizing interactions for S‐Q bond formation in Cpe0147. Figure S6. Distance distributions of the interactions made by catalytically relevant residues in molecular dynamics simulations. Figure S7. Distributions of pK a and percentage buried for catalytically relevant residues in molecular dynamics simulations. Figure S8. Convergence of the free energy values along χ1 and χ2 in metadynamics simulations. Figure S9. Block error analysis of free energy across collective variables in metadynamics simulations. Figure S10. Errors associated with free energy surfaces in metadynamics simulations. [file PRO-34-e70238-s001.pdf]

Supplementary Material

Protease mimicry: dissecting the ester bond crosslinking mechanics in bacterial adhesin proteins

Yuliana Yosaatmadja, Vanessa Ung, Xinlu Liu, Yixuan Zhao, Julia K. Wardega, Aria Shetty, Sophie Schoensee, Ivanhoe K. H. Leung, Jeremy R. Keown, David C. Goldstone, Edward N. Baker, Paul G. Young, Davide Mercadante, Christopher J. Squire.

Genus species	UniProt Accession and aa sequence	PDB ID*	RMSD[#] (Å)	Sequence Identity[#] (%)
Clostridium perfringens	A0AAV3BQK0<br>298 – 438	4NI6	0.00	100
Clostridium perfringens	A0AAV3BQK0<br>292 – 438	4MKM (domain1)	0.59	100
Clostridium perfringens	A0AAV3BQK0<br>439 – 587	4MKM (domain 2)	0.63	85
Suipraoptans intestinalis	A0A6N7UZI9<br>883 – 1031	8F90	0.98	44
Enterococcus columbae	S1N325<br>502 – 647	7UI8	1.06	48
Gemella bergeri	U2Q1B9<br>731 – 883	7UC3	1.10	44
Suipraoptans intestinalis	A0A6N7UZI9<br>623 – 772	8FHA	1.12	46
Gemella massiliensis	WP_327058535[†]<br>340 – 489	8F9L	1.32	44
Mulieris mulieris	A0A7X9TCH6<br>6668 – 6959	5U5O	1.87	22

* All proteins display the same Ig-like domain topology. [#] Relative to WT structure, *C. perfringens* PDB ID 4NI6. [†] NCBI Reference Sequence.

Supplementary Table S1. Structural and sequence comparison of ester bond adhesin domains released in the Protein Data Bank.

	T450C	T450S
PDB identifier	9BLO	9BLP
**Data Collection and processing:**		
Diffraction source	MX1, Australian Synchrotron	MX1, Australian Synchrotron
Wavelength (Å)	0.9537	0.9537
Space group	*P*1	*C*2
Cell dimensions:		
a, b, c (Å)	39.98, 44.40, 50.1	54.91 44.17 64.70
α, β, γ (°)	95.1, 109.4, 110.1	90.00 110.1 90.00
Resolution (Å)*	18.86 – 1.35 (1.37 – 1.35)	17.87 – 1.20 (1.22 – 1.20)
CC1/2*	1.000 (0.79)	0.999 (0.95)
$I/\sigma(I)$*	27.8 (2.4)	20.2 (3.8)
Completeness (%)*	94.8 (77.4)	97.7 (93.9)
Multiplicity*	3.3 (3.3)	7.1 (6.8)
**Refinement:**		
Resolution (Å)	18.86 – 1.35	17.87 – 1.20
No. of reflections	58614	42278
R_{work}	0.193	0.142
R_{free}	0.229	0.158
No. of Atoms:		
Protein	Chain A, 1163 Chain B, 1157	2989
Water	283	144
Metal	4	3
Glycerol	---	28
Average *B*-factor	16.1	19.3
**Validation**		
RMSD bond lengths (Å)	0.005	0.007
RMSD bond angles (°)	1.19	1.34
MolProbity score (percentile)	100th	98th
Ramachandran favoured (%)	99	98

*Data in parentheses is for the high-resolution shell.

Supplementary Table S2. Data collection, refinement, and validation statistics for T450C and T450S X-ray crystal structures

Simulation type	System	Number of replicates	Sampling per replicate (ns)
Equilibrium molecular dynamics	Wild type	5	250
	D577H	5	250
	T450S	5	250
	T450S/H572E	5	250
Metadynamics	Wild type	3	6000
	T450S	3	6000

Supplementary Table S3. Molecular dynamics and metadynamics replicate and sampling parameters.

Distance between	P-value	Test statistic	Critical values at given confidence level		
			1%	5%	10%
T450 – H572	$2.66 x 10^{-20}$	-11.170	-3.430	-2.862	-2.567
S450 – H572	$7.73 x 10^{-14}$	-8.581			
T450 – D577	$1.27 x 10^{-13}$	-8.497			
S450 – D577	$3.66 x 10^{-5}$	-4.889			
H572 – D577 (T/Q)	$2.04 x 10^{-30}$	-18.678			
H572 – D577 (S/Q)	$6.42 x 10^{-13}$	-8.221			
Q580:OE1 – D480:HD2 (T/Q)	$6.23 x 10^{-21}$	-11.440			
Q580:HE21/HE22 – D480:OD1 (T/Q)	$2.254 x 10^{-4}$	-4.467			
Q580:OE1 – D480:HD2 (S/Q)	$1.409 x 10^{-4}$	-4.579			
Q580:HE21/HE22 – D480:OD1 (S/Q)	$2.83 x 10^{-5}$	-4.947			

Supplementary Table S4. Unit-root test statistics for time traces of the distances between mechanistically relevant residues. The presence of unit roots in the distance time series obtained for equilibrium simulations of WT Cpe0147 and T450S mutant Cpe0147 (Figure S6) were assessed using Augmented-Dickey Fuller tests. Each timeseries contains 100,000 data points. The null hypothesis assumes that a unit root is present but can be rejected in favour of the alternative hypothesis (no unit root), if the *P*-value is below the critical value at a given confidence level. The more negative the test statistic the stronger the rejection of the null hypothesis at a given confidence level. Critical values at confidence levels of 1%, 5% and 10% have been taken from previously tabulated test statistics.[64]

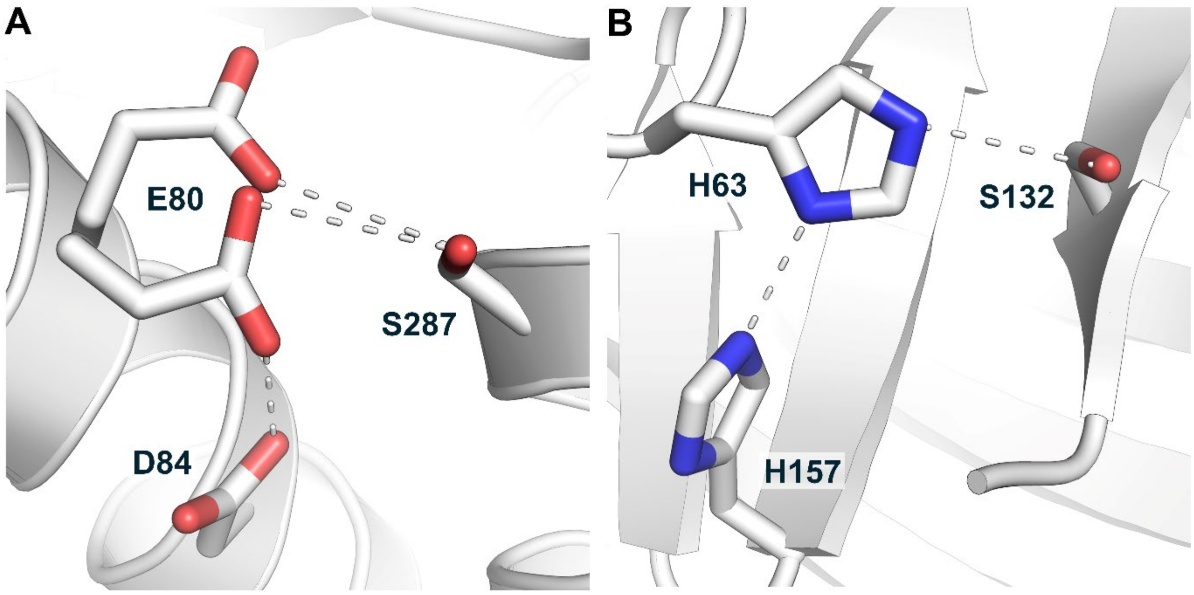

Figure S1. The active site triads of two non-cannonical serine proteases. A. Sedolisin, a serine protease from *Pseudomonas sp.* (PDB ID 1KDV), substitutes the canonical histidine base of a serine protease with a glutamate (E80). **B.** The *Human β-herpesvirus 5* protease (PDB ID 1CMV) has a histidine, H157, in the classical acid position of the serine protease catalytic triad. This protease is also functional with a simpler dyad of H63/S132.

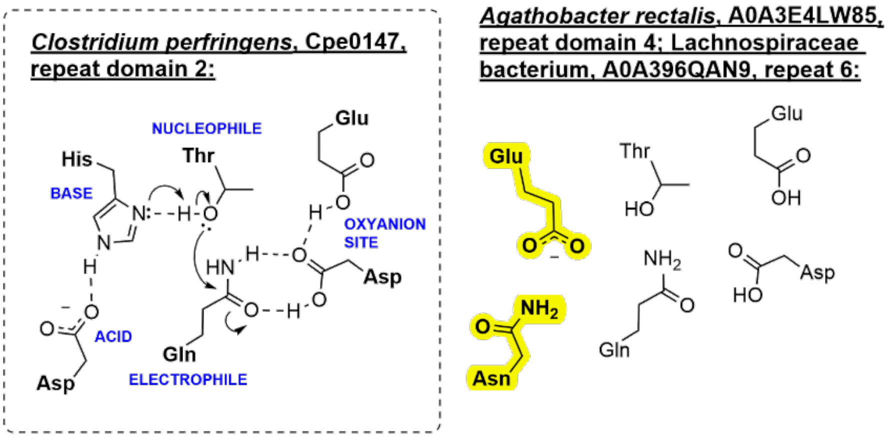

Protein:	UNIPROT Code:	Role and structure:	Taxonomy:*	Structural Source:	Catalytic machinery:	
Agathobacter rectalis, A0A3E4LW85 repeat domain 4 [Pro596-Pro719]	A0A3E4LW85	VaFE repeat-containing surface-anchored protein, mixed isopeptide & ester repeat domains (total 8)	Bacillota, Clostridia, Eubacteriales, Lachnospiraceae, *Agathobacter*, *A. rectalis*	AlphaFold	Acid<br>Base<br>Nucleophile<br>Electrophile<br>Oxyanion site	Asn<br>Glu<br>Thr<br>Gln<br>Asp, Glu
Lachnospiraceae bacterium, A0A396QAN9, repeat domain 6 [Pro730-Ile850]:	A0A396QAN9	VaFE repeat-containing surface-anchored protein, mixed isopeptide & ester repeat domains (total 7)	Bacillota, Clostridia, Eubacteriales, Lachnospiraceae, unclassified Lachnospiraceae	AlphaFold	Acid<br>Base<br>Nucleophile<br>Electrophile<br>Oxyanion site	Asn<br>Glu<br>Thr<br>Gln<br>Asp, Glu

* Phylum, Class, Order, Family, Genus, Species

Figure S2. Naturally occurring variations in the putative catalytic residues of Cpe0147-like ester crosslink domains. The identification of possible autocatalytic residues was achieved by visual inspection of X-ray crystal structures and AlphaFold predicted models overlaid with the Cpe0147 domain, PDB ID 4NI6. Residues that deviate from the canonical *Clostridium perfringens*, Cpe0147 adhesin domain 2 configuration, are highlighted in yellow.

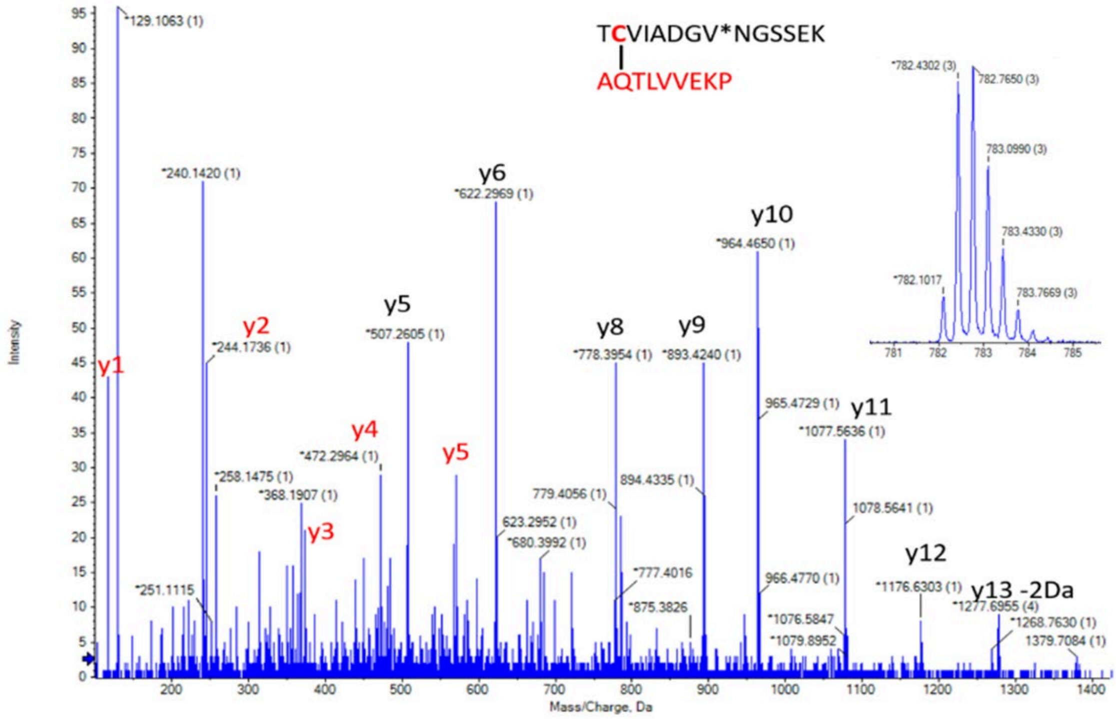

Figure S3. Trypsin digest coupled with tandem mass spectrometry for the T450C mutant. MS/MS spectrum of the m/z 782.77 3+ ion, representing the cross-linked peptides TCVIADGV*NGSSEK and AQTLVVEKP, with loss of ammonia (* indicates deamidated asparagine, +0.9848 Da). Charge states are indicated in parentheses. Inset shows the precursor ion spectrum.

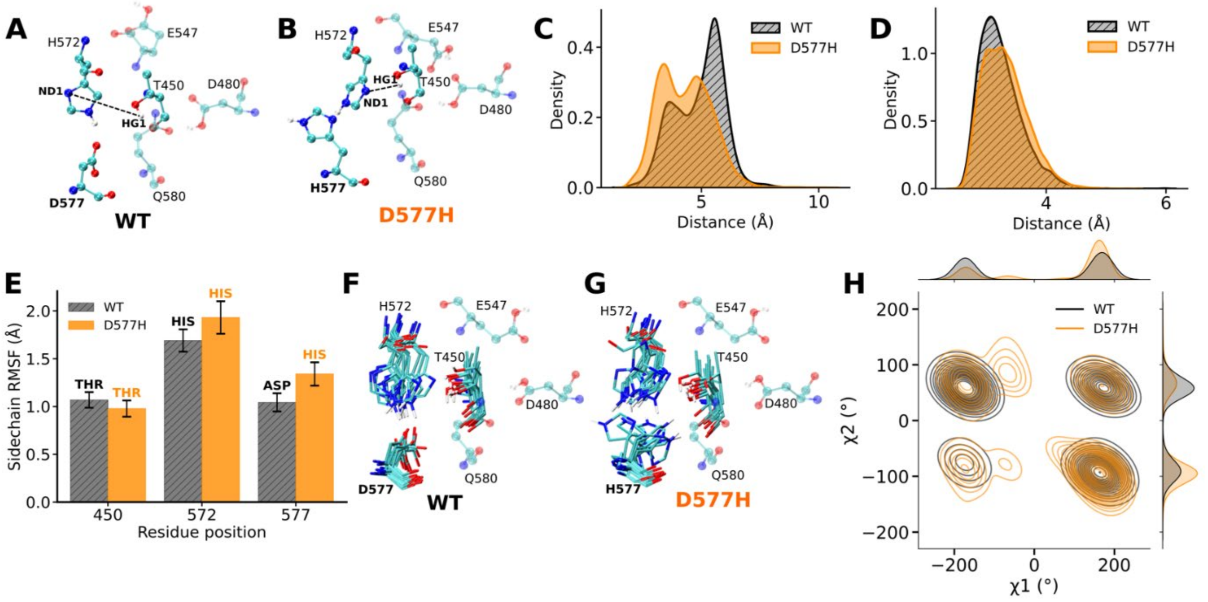

Figure S4: Stabilising interactions for T-Q bond formation in Cpe0147. A-B. Representative conformations of WT (**A**) and D577H Cpe0147 mutant (**B**). The black dashed line indicates the distance between H572:Nδ1 and T450:Hγ1, suggested within the mechanism of bond formation. **C-D**. Probability density distributions of the H572:Nδ1—T450:Hγ1 and T450:Oγ1—Q580:Nε2 distances, as sampled in MD simulations. **E**. Root mean square fluctuations (RMSF) of the residues suggested to influence the T-Q bond formation in the WT (grey) and D577H (gold) Cpe0147 constructs. **F-G**. Conformational ensembles of residues H572, D/H577 and T450, obtained from equilibrium molecular dynamics (MD) simulations of WT or D577H Cpe0147 constructs. **H.** Probability density function for H572 χ1 and χ2 dihedral angles, obtained from equilibrium simulations of WT (grey) and D577H (gold) Cpe0147 constructs.

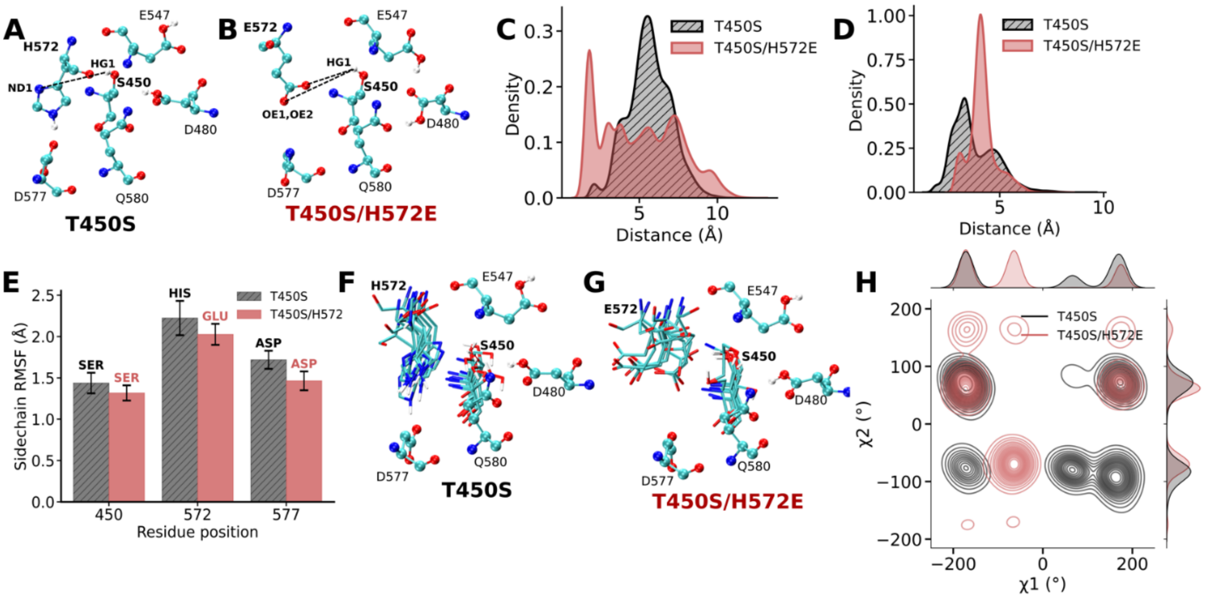

Figure S5: Stabilising interactions for S-Q bond formation in Cpe0147. A-B. Representative conformations of T450S (**A**) and T450S/H572E Cpe0147 mutants (**B**). The black dashed line indicates the distance between H572:Nδ1 and S450:Hγ1 (**A**), or E572:Oε1,Oε2 and S450:Hγ1 (**B**), suggested within the mechanism of bond formation. **C-D.** Probability density distributions of the H572:Nδ1—S450:Hγ1 and S450:Oγ1—Q580:Nε2 distances, as sampled in MD simulations. **E.** Root mean square fluctuations (RMSF) of the residues suggested to influence the Ser-Gln bond formation in the T450S (grey) and H572E (pink) Cpe0147 constructs. **F-G.** Conformational ensembles of residues H/E572, D577 and S450, obtained from equilibrium molecular dynamics (MD) simulations of T450S or T450S/H572E Cpe0147 constructs. **H.** Probability density function for H572 and E572 χ1 and χ2 dihedral angles, obtained from equilibrium simulations of T450S (grey) and H572E (pink) Cpe0147 constructs.

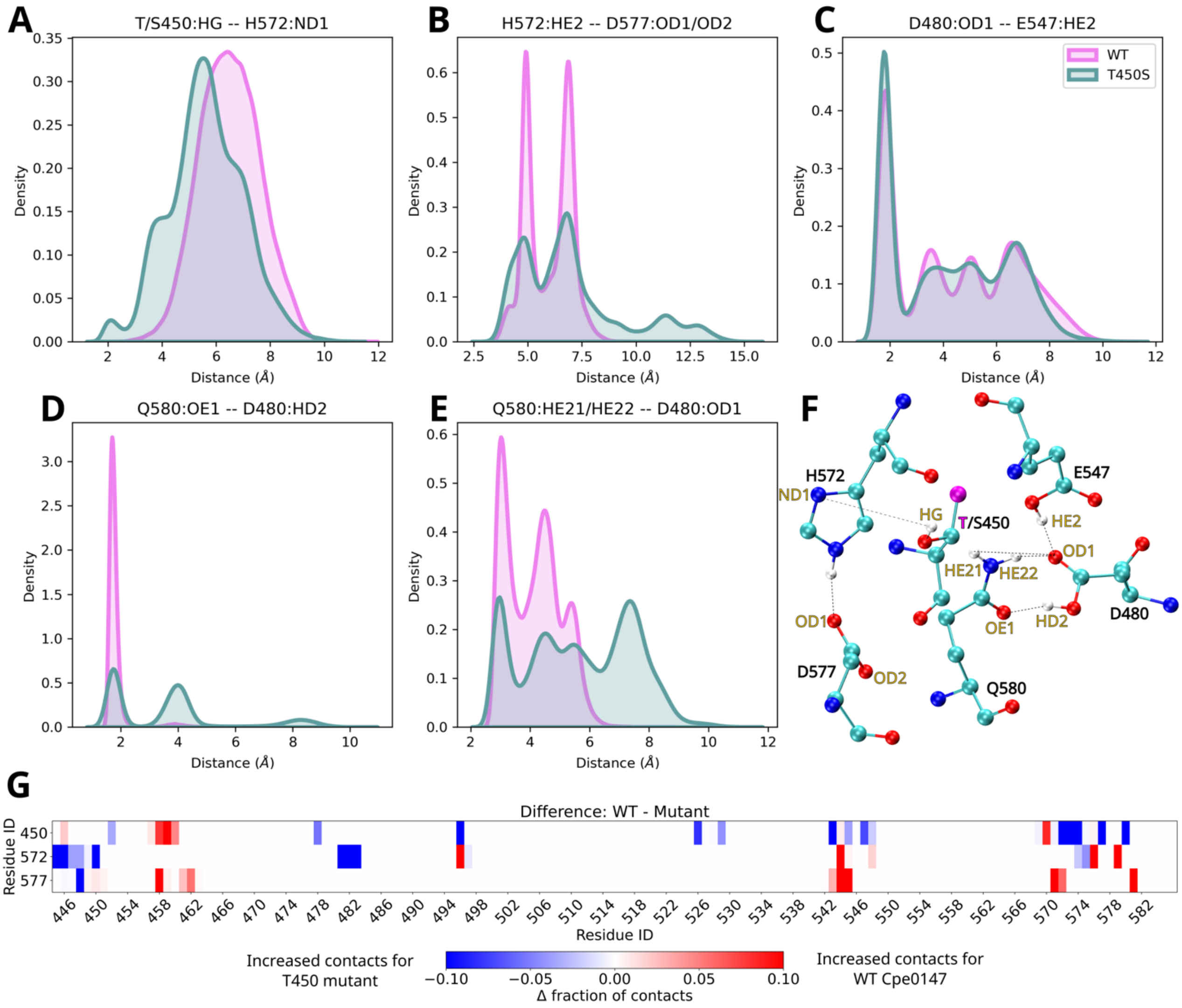

Figure S6: Distance distributions of the interactions made by catalytically relevant residues in molecular dynamics simulations. A-E. Distance distributions obtained from equilibrium molecular dynamic simulations of WT Cpe0147 (pink) and the T450S mutant (grey), for hydrogen bonds formed between catalytically relevant residues illustrated in **F** – specifically, the distances between: Hγ of residue 450 and Nδ1 of H572 (**A**), Hε2 of H572 and Oδ1/ Oδ2 of D577 (**B**), Oδ1 of D480 and Hε2 of E547 (**C**), Oε1 of Q580 and Hδ2 of D480 (**D**), and Hε21/Hε22 of Q580 and Oδ1 of D480 (**E**). **F.** Representative conformations of the catalytically relevant residues within WT Cpe0147 and the T450S mutant. The magenta bead represents the methyl group in the side chain of T450 in WT Cpe0147. Dashed lines indicate the distances between residues reported in panels **A-E**. Residues are labelled in black and their respective atoms of interest are labelled in yellow. **G.** Contact map (calculated at a cutoff of 4.5 Å) of the difference between the fraction of contacts formed by WT Cpe0147 and the T450S mutant, against residues 450, 572 and 577 across the simulation time. Positive values indicate a higher fraction of contacts formed for WT Cpe0147 while negative values indicate a higher fraction of contacts formed for the T450S mutant.

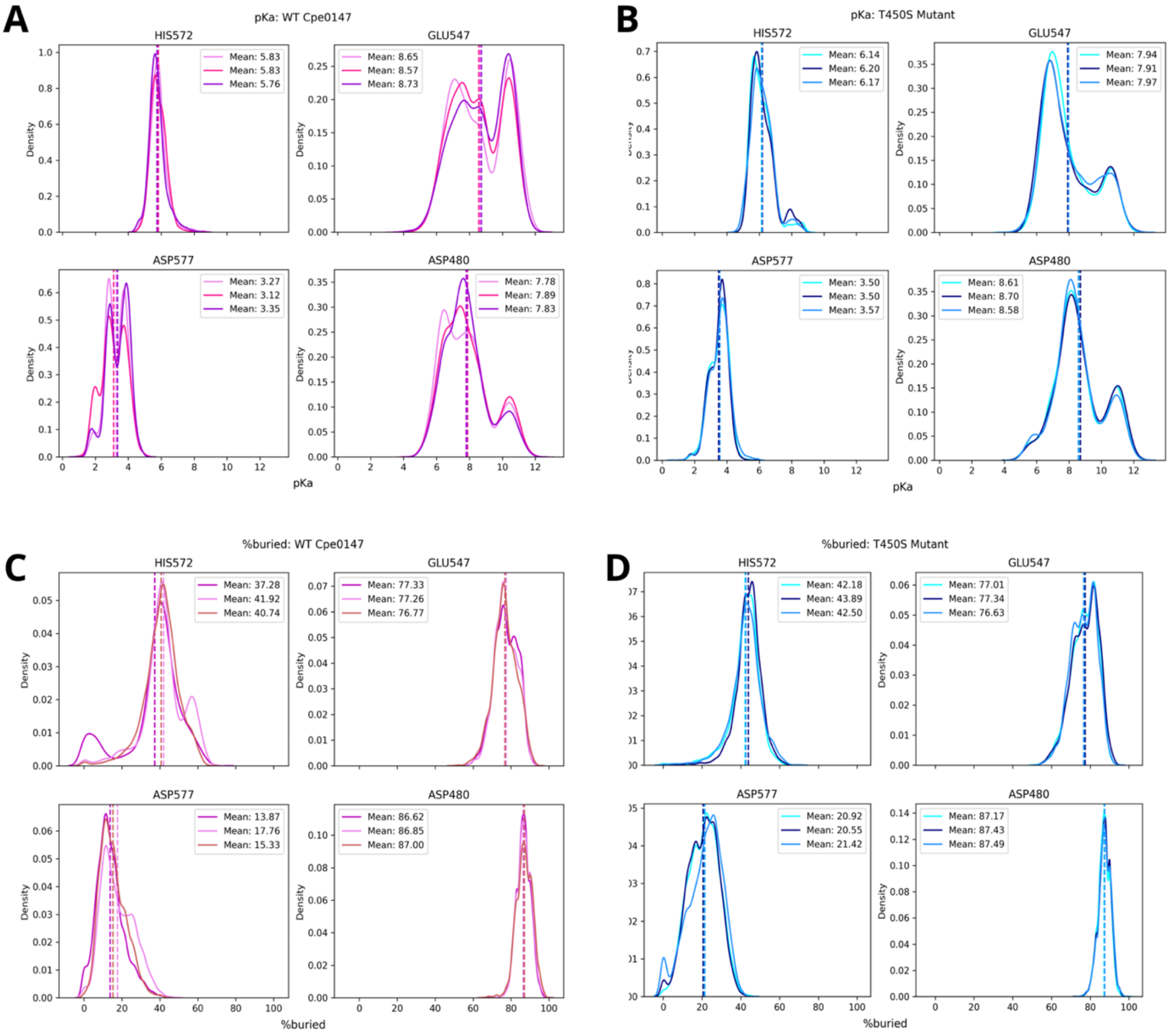

Figure S7: Distributions of pK_a and percentage buried for catalytically relevant residues. A-B. pK_a distributions for residues H572, E547, D577 and D480 in WT Cpe0147 (**A**) and the T450S mutant (**B**) obtained from each replicate of the metadynamics simulations. Dashed lines indicate mean pK_a values reported in the legend. **C-D.** Percentage buried distributions for residues H572, E547, D577 and D480 in WT Cpe0147 (**C**) and the T450S mutant (**D**) obtained from each replicate of the metadynamics simulations. Dashed lines indicate mean percentage buried, also reported in the legend. pK_a values and percentage buried values were calculated using PROPKA3.[67,68]

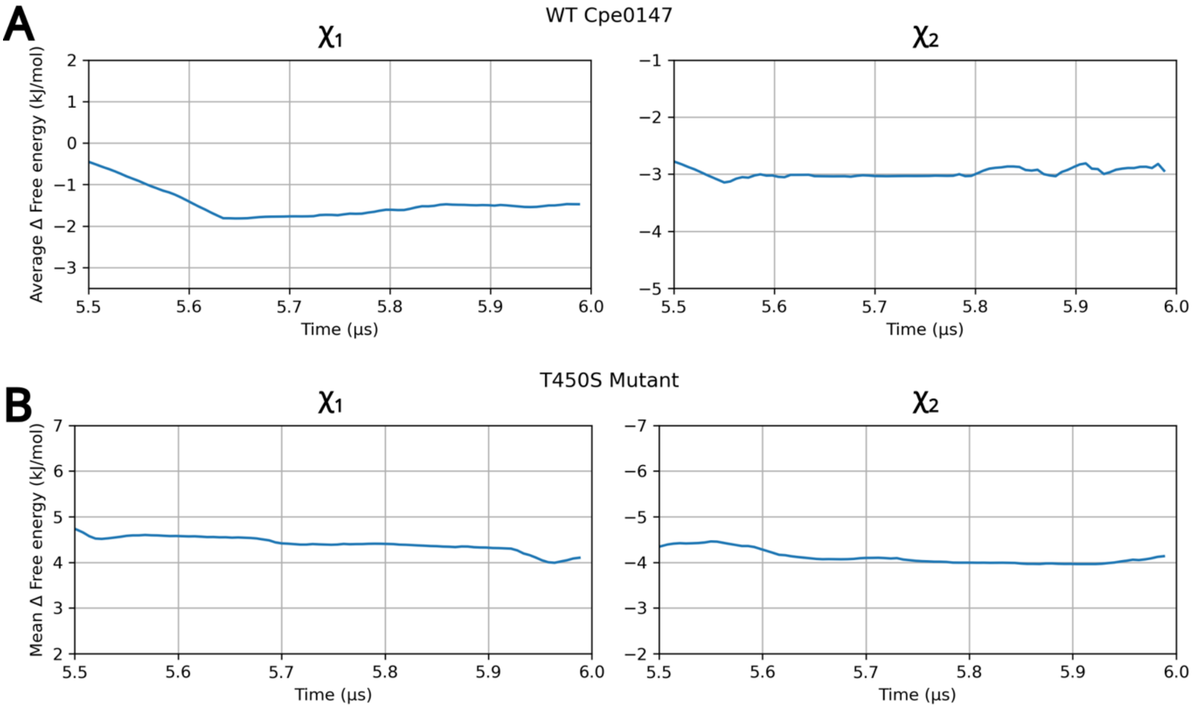

Figure S8: Convergence of the free energy values along χ1 and χ2. A-B. The mean free energy difference between energy minima along the collective variables χ_1 (left) and χ_2 (right), across replicates of the metadynamics simulations for WT Cpe0147 (**A**) and the T450S mutant (**B**) are shown as a function of the last 500 ns of simulation time.

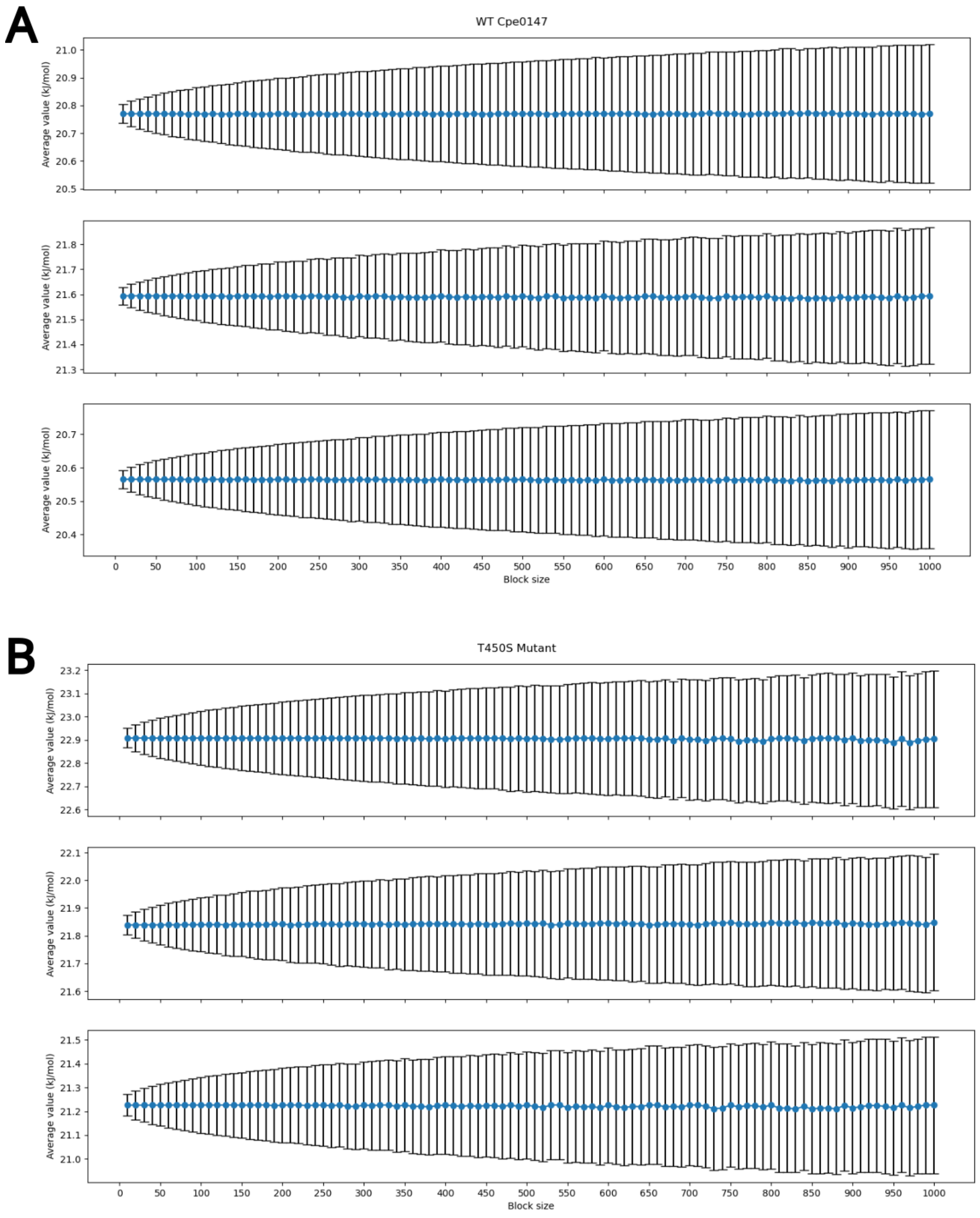

Figure S9: Block error analysis of free energy across collective variables in metadynamics simulations. A-B. Block averages of the free energy and associated standard deviations, obtained along the collective variables χ_1 and χ_2, reported as a function of block size for the metadynamics simulation replicates of WT Cpe0147 (**A**) and the T450S mutant (**B**).

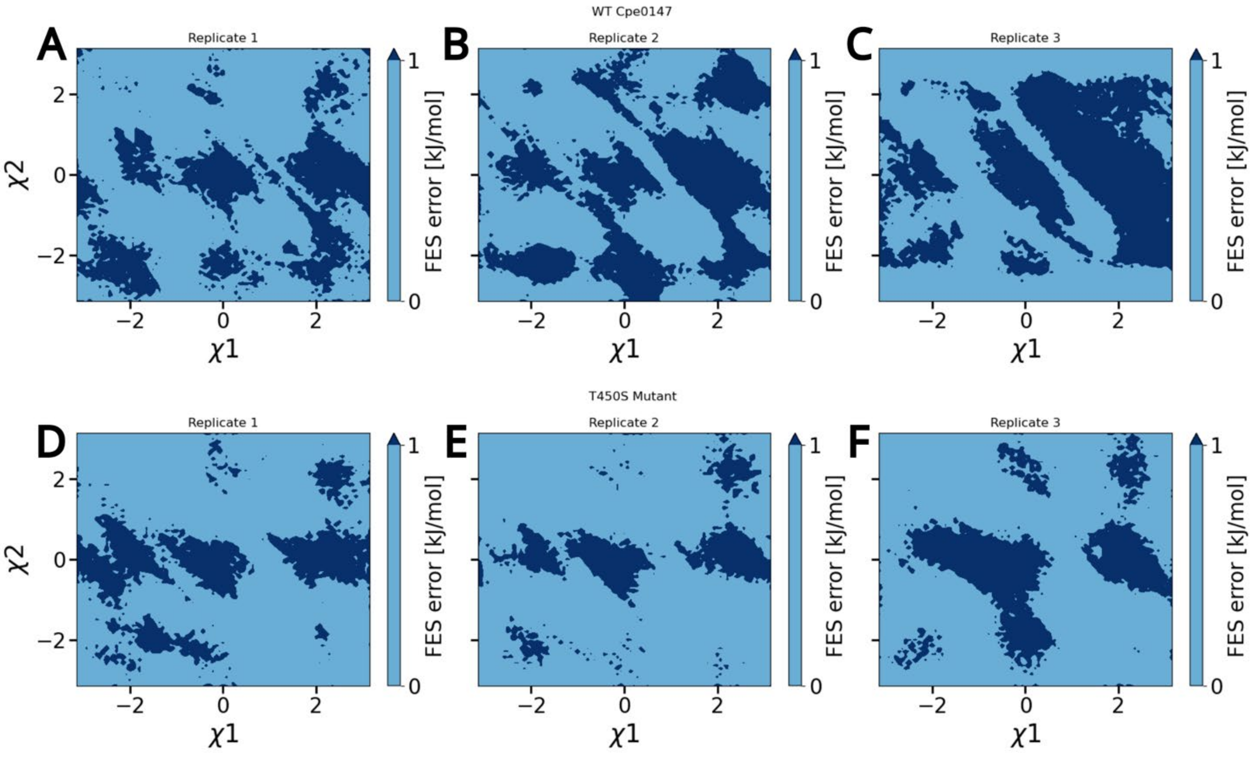

Figure S10: Errors associated with free energy surfaces in metadynamics simulations. A-F. Free energy surfaces reporting the error associated with the free energies along χ_1 and χ_2, shown in Figure 5, for metadynamics replicates of WT Cpe0147 (**A-C**) and the T450S mutant (**D-F**).
